# The conserved Pelado/ZSWIM8 protein regulates actin dynamics by promoting linear actin filament polymerization

**DOI:** 10.26508/lsa.202201484

**Published:** 2022-08-08

**Authors:** Claudia Molina-Pelayo, Patricio Olguin, Marek Mlodzik, Alvaro Glavic

**Affiliations:** 1 Department of Cell, Developmental, and Regenerative Biology, Graduate School of Biomedical Sciences, Icahn School of Medicine at Mount Sinai, New York, NY, USA; 2 Departamento de Biología, Centro FONDAP de Regulación del Genoma, Facultad de Ciencias, Universidad de Chile, Santiago, Chile; 3 Departamento de Neurociencia, Programa de Genética Humana, Instituto de Ciencias Biomédicas, Instituto de Neurociencia Biomédica, Facultad de Medicina, Universidad de Chile, Santiago, Chile

## Abstract

Actin filament polymerization can be branched or linear, which depends on the associated regulatory proteins. Competition for actin monomers occurs between proteins that induce branched or linear actin polymerization. Cell specialization requires the regulation of actin filaments to allow the formation of cell type–specific structures, like cuticular hairs in *Drosophila*, formed by linear actin filaments. Here, we report the functional analysis of CG34401/*pelado*, a gene encoding a SWIM domain–containing protein, conserved throughout the animal kingdom, called ZSWIM8 in mammals. Mutant *pelado* epithelial cells display actin hair elongation defects. This phenotype is reversed by increasing actin monomer levels or by either pushing linear actin polymerization or reducing branched actin polymerization. Similarly, in hemocytes, Pelado is essential to induce filopodia, a linear actin-based structure. We further show that this function of Pelado/ZSWIM8 is conserved in human cells, where Pelado inhibits branched actin polymerization in a cell migration context. In summary, our data indicate that the function of Pelado/ZSWIM8 in regulating actin cytoskeletal dynamics is conserved, favoring linear actin polymerization at the expense of branched filaments.

## Abstract (precis)

Pldo/ZSWIM8 is a conserved protein that promotes linear actin polymerization at the expense of branched actin in several contexts, ranging from *Drosophila* morphogenesis to human cell migration.

## Introduction

The regulation of actin dynamics is essential in each cell to establish cell shape and drive cell division, migration, or morphological changes of whole tissues. Actin polymerization is regulated by several proteins that bind to either monomeric or filamentous actin (Vitriol et al, 2015; and rev. in Revenu et al [2004], Campellone and Welch [2010], Bogdan et al [2013], Davidson and Wood [2016], Carlier and Shekhar [2017], Rottner et al [2017], and Hohmann and Dehghani [2019]). Over 150 proteins have been described to date, to participate as actin-binding proteins, promoting or inhibiting specific features of its dynamic polymerization (Luo et al, 1997; Mullins et al, 1998; Machesky et al, 1999; Zallen et al, 2002; Fricke et al, 2009; Ito et al, 2011; Chen et al, 2014; Gurung et al, 2016 and rev. in Frankel and Moosekert [1996], Goley and Welch [2006], Kovar [2006], Takenawa and Suetsugu [2007], Pollitt and Insall [2009], Fletcher and Mullins [2010], Rotty et al [2012], and Bogdan et al [2013]). Actin polymerization can be classified as either branched or linear, depending on the associated regulatory proteins (rev. in Campellone and Welch [2010] and Suarez and Kovar [2016]). Linear actin polymerization generally requires the activity of the formin protein family, like Diaphanous/Dia (rev. in Campellone and Welch [2010] and Bogdan et al [2013]), whereas branched polymerization requires the activation of the Arp2/3 complex, which is regulated among others, by nucleator promoting factors (NPFs) from the WASP family, including Scar/WAVE or Wasp (Mullins et al, 1998; and rev. in Frankel and Mooseker [1996], Le Clainche and Carlier [2008], Campellone and Welch [2010], Jockusch [2017], and Gautreau et al [2022]). Although cells have a high concentration of actin monomers (Koestler et al, 2009; Burke et al, 2014; and rev. in Pollitt and Insall [2009]), most of them are sequestered by different regulatory proteins, establishing a competition for actin monomers between the two distinct forms of polymerization (Burke et al, 2014; Henson et al, 2015; Suarez et al, 2015; and rev. in Suarez and Kovar [2016]). In most cells, there are a larger number of proteins that induce branched actin polymerization, and thus, competition for actin monomers is often tilted toward branched polymerization arrangements (Suarez et al, 2015). Profilin (*chickadee/chic* in *Drosophila*) is pivotal in the polymerization selection process with Chic/Profilin both favoring linear actin polymerization, mediated by the formin protein family, and also inhibiting branched polymerization by preventing the binding of NPFs to actin monomers (Suarez et al, 2015).

Cell specialization requires actin cytoskeleton regulation to allow the formation of specific structures, like for example cuticular hairs in *Drosophila*. Cuticular epithelial cells in *Drosophila* form a single cuticular actin hair at the apico-distal vertex of each cell. Hair formation depends on the Frizzled planar cell polarity (Fz/PCP) pathway (rev. in Seifert and Mlodzik [2007], Vladar et al [2009], Adler [2012], Peng and Axelrod [2012], Carvajal-Gonzalez and Mlodzik [2014], Devenport [2014], Hale and Strutt [2015], Yang and Mlodzik [2015, Aw and Devenport [2017], Humphries and Mlodzik [2018], and Harrison et al [2020]), which controls the positioning and polarized actin accumulation by regulating the hierarchical activity of groups of genes that constitute it: core PCP genes (*frizzled/fz*, *disheveled/dsh*, *diego/dgo*, *Van gogh/Vang*, *prickle/pk*, and *flamingo/fmi*) and planar polarity effector (PPE) genes, which either inhibit actin polymerization at the proximal side of wing epithelial cells (e.g., *inturned*, *fritz*, *fuzzy*, and *multiple wing hairs/mwh*) or promote actin polymerization as positive effectors of the Fz/Dsh/Dgo complex (Axelrod et al, 1998; Shimada et al, 2001; Jenny et al, 2003, 2005; Das et al, 2004; Collier et al, 2005; Park et al, 2006; Wu et al, 2008; Wu & Mlodzik, 2008; Yan et al, 2008, 2009). One of these downstream effectors of the Fz/Dsh complex is RhoA (also known as Rho1 in *Drosophila*), which locally activates its effector proteins, including in this context the *Drosophila* Rho-associated kinase, Drok (Strutt et al, 1997; Winter et al, 2001). Drok activity focuses actin polymerization but does not affect the location or orientation of wing hairs (Winter et al, 2001; Yan et al, 2009; and rev. in Adler [2012], Gao [2012], Strutt and Strutt [2009], and Carvajal-Gonzalez and Mlodzik [2014]). Actin hair localization and formation is widely used as a proxy to evaluate the Fz/PCP pathway functionality (Mitchell et al, 1983; Lee & Adler, 2004; Lu et al, 2010; Gault et al, 2012; Sagner et al, 2012; Carvajal-Gonzalez et al, 2016; and rev. in Strutt and Strutt [2009], Adler [2012], Gao [2012], and Carvajal-Gonzalez and Mlodzik [2014]).

Actin hair formation also requires the activity of the Shavenbaby/Shavenoid pathway that regulates the actin cytoskeleton (Delon et al, 2003; Ren et al, 2006). Because the hair structure, trichome, is formed by linear actin polymerization, it is a useful and well-characterized model system to identify and analyze proteins that regulate actin dynamics (Eaton et al, 1996; Turner & Adler, 1998; Winter et al, 2001; Guild et al, 2005; He et al, 2005; Ren et al, 2007; Yan et al, 2009; Adler et al, 2013; Matis et al, 2014; Adler, 2018). In particular, the wing epithelial cells in *Drosophila* are uniquely suited for such analyses (Mitchell et al, 1983; Klein et al, 2006; Sagner et al, 2012; and rev. in Strutt and Strutt [2009], Adler [2012], and Singh and Mlodzik [2012]). As the trichome is present in every cell and requires a specific and exquisite interplay of regulatory factors of actin dynamics, it serves as an excellent model to identify and investigate novel genes required in this process (Eaton et al, 1996; Turner & Adler, 1998; Guild et al, 2005; He et al, 2005; Ren et al, 2007; Fang & Adler, 2010; Adler et al, 2013; Fagan et al, 2014; Adler, 2018). Importantly, trichome formation is critically linked to linear actin polymerization and the associated formin, Diaphanous (Dia) (Mitchell et al, 1983; Lu & Adler, 2015). Despite accumulating knowledge of the mechanisms and proteins regulating polarized actin accumulation during trichome formation, many open questions remain, and particularly, the mechanisms regulating linear versus branched actin polymerization in the cuticular epithelial cells, and in other contexts, remain largely unknown (Turner & Adler, 1998; Delon et al, 2003; Guild et al, 2005; He et al, 2005; Ren et al, 2005, 2007; Fang & Adler, 2010; Fang et al, 2010; Gault et al, 2012).

Here, we describe the identification and functional characterization of CG34401/*pelado* (*pldo*), a gene encoding a multidomain protein that is conserved throughout the animal kingdom, called ZSWIM8 in mammals (Shi et al, 2020; the gene named *dorado* in this case). We show that it is required for the formation of the actin-based trichome formation in *Drosophila* cuticle cells, most notably on wings and the notum. This gene has also been characterized in *Caenorhabditis elegans*, as an E3 ubiquitin ligase, named EBAX-1, and is part of a ubiquitin ligase complex that includes elongins B and C and Cullin2 (Wang et al, 2013). Although this E3 ligase function was also described in specific cell lines (Shi et al, 2020), our data suggest that in the context of actin dynamics, Pldo/ZSWIM8 may act independently of its E3 ligase activity.

Our functional genetics and epistasis analyses demonstrate that Pldo favors linear actin polymerization–at the expense of branched polymerization–in *Drosophila* and human A549 cell line and thus that this function of Pldo in regulating the actin dynamics is conserved. Immunoprecipitation assays suggest that Pldo might be part of a multiprotein complex that includes Scar/WAVE, Diaphanous, and Profilin. Structure function studies reveal that the N-terminal region of Pldo, containing the highly conserved SWIM, FH1, and WH2 domains, is sufficient to promote the formation/induction of filopodia in general and that the C-terminal part might be required for cell type–specific actin-related functions, for example, in epithelial cells.

Noteworthily, the *pelado* gene was also identified in a screen related to human diseases (microcephaly) (Yamamoto et al, 2014), without a described mechanistic function in that context, which makes it a very interesting protein to analyze. Consistent with our data on actin regulation established below, *pldo* function in the central nervous system might be related to axon/dendrites formation or radial glial migration that give rise to the neurons that populate the different brain layers, being all processes that requires specific actin polymerization regulation (Kullmann et al, 2020; and rev. in Rust et al [2012], Cooper [2013], and Chou and Wang [2016]). Taken together, our studies suggest that *pldo* is an essential regulator of actin dynamics in *Drosophila* and human cells, with a likely link in the disease context and even potential roles related to neurodegenerative diseases.

## Results

### Pldo, a novel gene required for cellular hair formation in *Drosophila*

**Figure 1.**
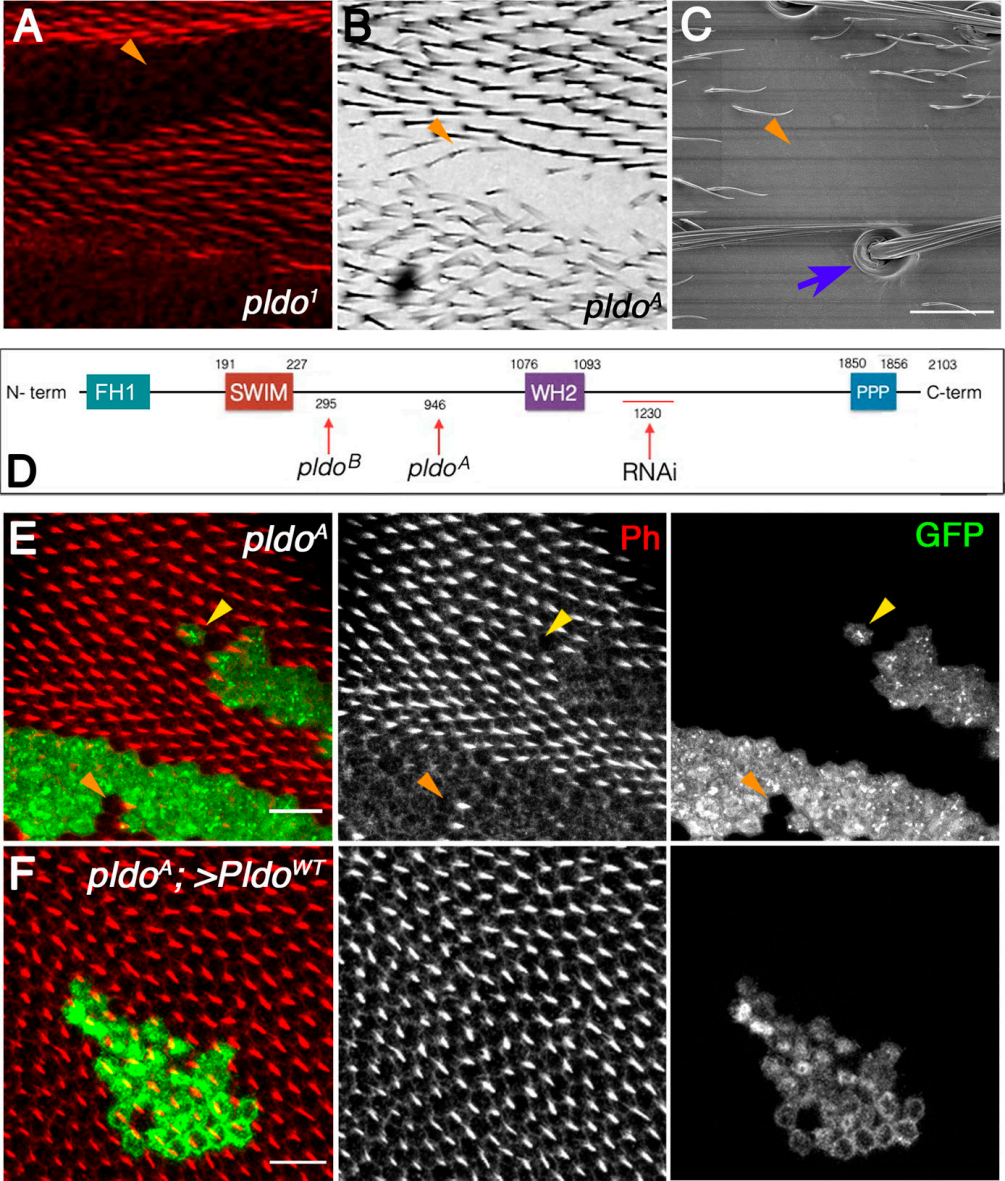
Pldo is required for epithelial cuticle hair formation. **(A, B, C, D)** General description of CG34401/*pldo*-mutant phenotypes and protein sequence. Unmarked *pldo*^*1*^ clones showing the absence of trichomes (actin hairs) in a pupal wing (A), adult wing (B, *pldo*^*A*^ clone), and adult thorax (C, SEM micrograph of thorax with *pldo*^*1*^ clone). **(A, B, C)** Scale bars correspond to 100 μm in (A) and 50 μm in (B) and (C). Note that cuticular cellular hairs are missing (orange arrowheads) but that sensory bristles are not affected (blue arrow). **(D)** Schematic presentation of the Pldo protein sequence (D) with the main conserved domains indicated (residue numbers are above the protein sequence line). Below the line are the characterized mutant alleles indicated, *pldo*^A^ and *pldo*^B^ (available in the Bloomington Stock Center), all showing the same phenotypes. These two alleles have point mutations: *pldo*^A^: G18874432A generates a nonsense mutation (W946Stop codon); and *pldo*^B^: C18872412T generating the change T295M. *pldo*^1^ is not indicated because its mutation has not been identified. The RNAi construct sequence (BL 18553) is also shown. **(E)** Pupal wings with MARCM *pldo*-mutant clones (mutant cells marked in green with GFP). Cellular hairs were visualized by rhodamine phalloidin staining (red). Note that the *pldo* LOF phenotype is fully cell autonomous, as observed in either a single-cell clone (yellow arrowhead) or a two-cell wild-type patch (orange arrowhead) displaying the absence or presence of hairs, respectively. Scale bars correspond to 50 μm. **(F)** *pldo* loss-of-function hair phenotype is fully rescued by the expression of Pldo^WT^ (via UAS in the MARCM clones), with hairs showing normal polarity and length. Scale bar represents 50 μm.

**Figure S1.**
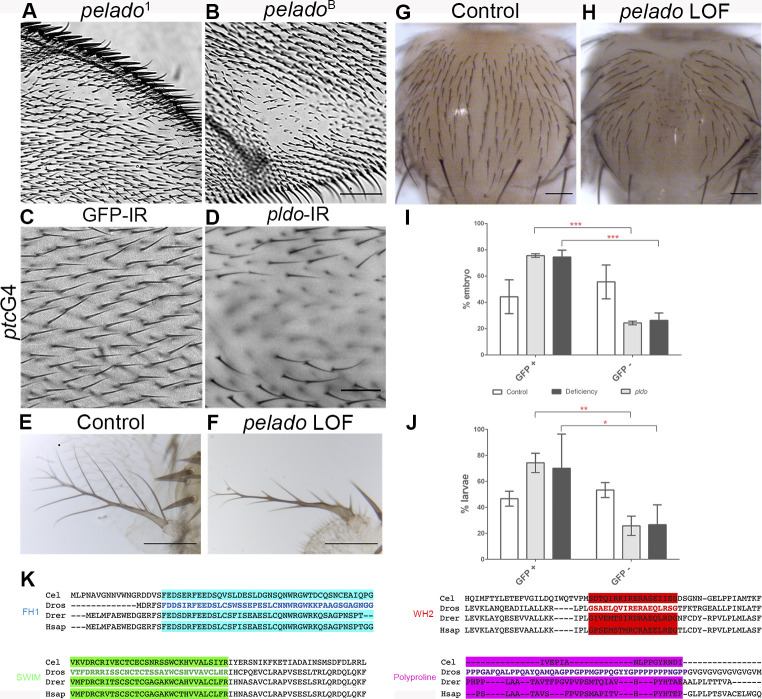
Pldo is required for hair and laterals formation. **(A, B)** Characterization of CG34401/*pldo* LOF phenotypes. Adult wings with unmarked *pldo* clones showing the absence of hairs phenotype. In these cases, different alleles for *pldo* were used (*pldo*^1^ and *pldo*^B^). Scale bars correspond to 100 μm. **(C, D)** Adult wings showing the *pldo* LOF phenotype generated by the expression of RNAi (and RNAi against GFP as a control) in the *ptcGal4* expression domain in the wing (note that *ptc* is expressed between veins 2 and 3 in the wing along the A/P compartment boundary, see schematic of *ptc* expression in Fig 3A). Scale bars correspond to 50 μm. **(E, F)** *pldo* LOF phenotype in adult antenna, generated by *ey-Gal4* driven RNAi expression (and control RNAi for comparison). Scale bars correspond to 50 μm. **(G, H)** Adult thorax showing the absence of cuticular hairs generated by the expression of RNAi (and RNAi against GFP as a control) in the *pannierGal4* expression domain in the notum (note that bristles are not affected). The scale bar corresponds to 100 μm. **(I, J)** Quantification of embryo and larvae mutants for *pldo* (GFP negative) or control (GFP positive). In each experiment, at least 20 embryo/larvae were considered per genotype, n = 3. Two-way ANOVA plus Sidak’s multiple comparison test was used for statistical analysis, **** indicates *P* < 0.0001; ** indicates *P* = 0.0039; and * indicates *P* = 0.0259. **(K**) *pldo* is located on the X chromosome at position X: 18,863,950… X: 18,884,075, and BLAST analysis indicates that *pldo* is conserved from *Caenorhabditis elegans* to humans. Protein sequence alignment of main predicted domains in Pldo from different species: *Cel*: *C. elegans*, *Dros*: *Drosophila*, *Drer*: *Danio rerio*, and *Hsap*: *Homo sapiens*. Alignment was obtained through the software package Clustal Omega (RRID:001591).

In an ENU mutant suppressor genetic screen, designed to identify the gene responsible for a wing growth phenotype produced by the overexpression driven by an EP line (EP361, [Cruz et al, 2009]), we generated a recessive embryonic lethal mutation on the X chromosome. To study the cellular phenotype in homozygous mutant cells, we used the Mosaic Analysis with a Repressible Cell Marker (MARCM) technique (Wu & Luo, 2006). Strikingly, homozygous mutant epithelial cells from different adult tissues, including the wing and notum, displayed a lack of the trichome (cellular actin–based hairs) phenotype (Fig 1A–C; also Fig S1A, B, G, and H). To identify the affected locus, we employed first a complementation strategy using several genomic duplications spanning the X chromosome, followed by small deficiencies to further restrict the chromosomal region associated with the phenotype. Subsequently, we knocked down the expression of candidate genes present within this chromosomal region by using dsRNA constructs and screened for the absence of trichomes, as seen in the phenotype obtained in homozygous mutant wing cells (Fig S1C, D, G, and H). This approach identified the previously uncharacterized gene CG34401 (specific target region of the RNAi construct is shown in Fig 1D) as responsible for the “loss-of-hairs” phenotype. As the defect associated displayed a hairless, or “bald,” phenotype, we named this gene *pelado* (*pldo*, which is the Spanish word for “bald”).

The CG34401/*pelado* gene (*pldo*) is located on the X chromosome at position 17F2 and encodes a large protein of 2103 amino acids, approx. 250 kD in size (Fig 1D). In silico analysis of the Pldo sequence (see the Materials and Methods section) revealed the presence of several conserved domains (Figs 1D and S1). The SWIM domain (Swi2/SNF2 and MuDR zinc-binding), which is defined as a domain that can interact with both DNA and other proteins (Ko et al, 2014; Godin et al, 2015), gives the name to the human ortholog of Pldo, ZSWIM8 (Zinc finger SWIM-type containing 8; www.flybase.org). Interestingly, other conserved domains present in Pldo include the FH1 (formin homology 1 domain), a Wiskott–Aldrich homology 2 domain (WH2), and a polyproline region (Fig 1D, also Fig S1K), which are also present in several proteins that regulate actin cytoskeleton dynamics, including Scar/WAVE and the formin Diaphanous/Dia (Watanabe et al, 1997; Miki et al, 1998; and rev. in Paunola et al [2002] and Carlier et al [2013]). In particular, the WH2 domain binds to monomeric actin (rev. in Paunola et al [2002]), and FH1 and polyproline domains can bind to Profilin (Chicadee/Chic in *Drosophila*) (rev. in Faix and Grosse [2006]).

After the identification of *pldo* as the gene of interest (the specific location of the mutation in the *pldo*^*1*^ allele is unknown), we identified two additional mutant alleles via complementation analyses. These alleles were generated in a genetic screen in the lab of Hugo Bellen intended to look for mutants related to human diseases (Haelterman et al, 2014; Yamamoto et al, 2014; both available in the Bloomington Drosophila Stock Center/BDSC with stock numbers 52333 and 52334). We subsequently refer to these molecularly characterized alleles as *pldo*^*A*^ and *pldo*^*B*^, respectively (Fig 1D), with one being a nonsense mutation that produces a stop codon at residue 946, W946STOP (*pldo*^*A*^), and the other a point mutation, T295M (*pldo*^*B*^; see Fig 1D for details). In accordance with the analysis of our original allele (*pldo*^*1*^), homozygous mutant clones of these characterized alleles also displayed loss of cellular hairs/trichomes (Figs 1E and S1A and B). This phenotype is cell-autonomous (see examples in Fig 1E, with very small clones displaying the respective features). To unequivocally confirm the identity of the gene responsible for the loss of trichomes, we generated a transgenic line to express the *pldo*^*WT*^ coding sequence in the *pldo*^A^ mutant background using the MARCM technique (Wu & Luo, 2006). Indeed, the expression of a *pldo*^WT^ transgene completely rescued the hair loss defects, with no other evident phenotype in the mutant clones (Fig 1F). Because these BDSC alleles are molecularly characterized and showed the same phenotype as our original allele, we used the *pldo*^A^ allele for further analyses.

As *pldo* is a recessive lethal mutation, we next wished to establish the stage of lethality. To determine this, we generated *pldo*-mutant stocks balanced with FM7 (marked with GFP expressed under *twist* control), and thus, embryos homozygous mutant for *pldo* can be identified as GFP-negative embryos or larvae. The number of GFP-negative embryos (*pldo* mutants) was considerably lower than that of controls. We were also able to observe a small number of mutant larvae (at 24 h AEL [after egg-laying]) with more than half of these being already dead, and those still alive showed severe motor defects (see summary of these results in Fig S1I and J). Taken together with maternal expression of *pldo* (www.flybase.org), these results suggest an important function of *pldo* during early embryonic developmental stages, which is obscured by maternal contribution.

### Pldo is required for actin hair formation and elongation

**Figure 2.**
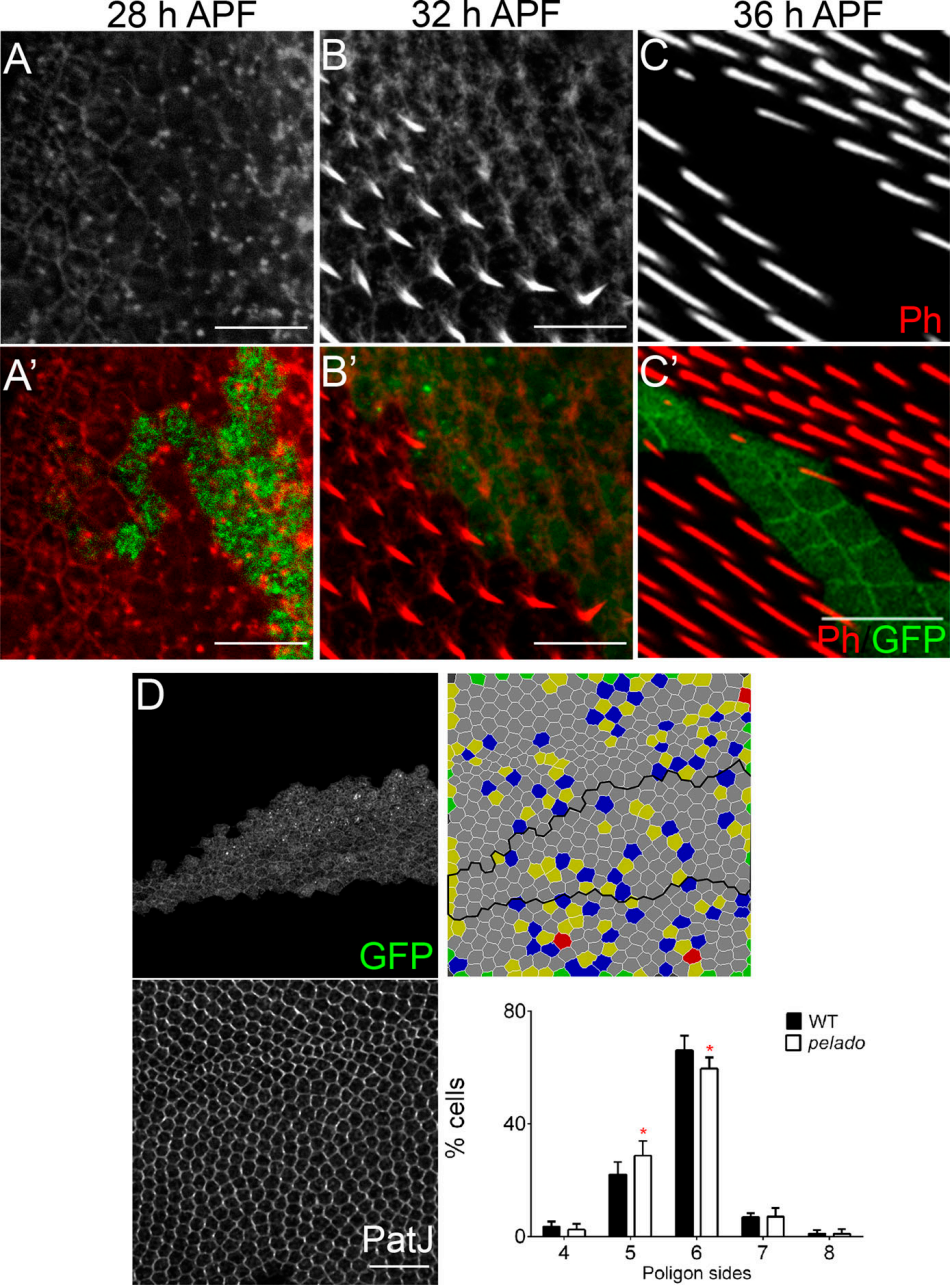
Pldo is required for actin hair formation and cell shape maturation. **(A, B, C)** Temporal characterization of the mutant *pldo* phenotype during cellular hair formation. Confocal micrographs showing *pldo* MARCM clones (marked by GFP, green) during actin hair formation (labeled via rhodamine phalloidin, Ph). **(A, B, C)** Monochrome images showing actin alone (Ph) and (A′, B′, C′) displaying clonal marker (green) and actin (red). Note the absence of hair elongation from 30 to 32 h APF onward, in mutant GFP marked cells. (A, A′) Note accumulation of actin appears normal in control and mutant cells at 28 h APF. All the images are oriented with distal in the lower right corner. Scale bars correspond to 50 μm. **(D)** Evaluation of cell shape in *pldo*-mutant cells, at 28–32 h APF, as compared with neighboring wild-type cells. Note the significant reduction in the number of hexagonal cells in *pldo*-mutant clones (marked by GFP; cell outline displayed via PatJ staining, a junctional marker). Six different individuals were analyzed, with around 100 cells in each case. *P* = 0.0366 via *t* test. Scales bar correspond to 100 μm.

Actin hair formation in cuticular epithelial cells takes place in three stereotyped stages: apical actin accumulation, prehair formation, and hair elongation (Guild et al, 2005). Each stage is characterized by specific actin distributions and hair features. At around 28 h after puparium formation (APF), actin starts accumulating apically in the distal vertex of every epithelial wing cell (Mitchell et al, 1983; Turner & Adler, 1998). At 32 h APF, the structure called “prehair” appears, and finally at 36 h APF, the hair is almost completely formed or elongated (Classen et al, 2008; all time points relate to a growing temperature of 25°C). To address the potential role of Pldo in these stages, we generated *pldo*-mutant clones at third instar larvae and followed the cellular maturation and hair growth in the wing epithelia at the time points of pupal development mentioned above. Although at 28 h APF there were no obvious differences in actin distribution between *pldo*-mutant clones (GFP-positive cells) and control cells (Fig 2A), clear alterations in actin distribution and defects in (pre)hair formation were observed at 32 and 36 h APF (Fig 2B and C). In *pldo*-mutant cells, the prehair formation process was impaired, and defects were most evident from early stages of hair elongation. Thus, the core PCP-regulated hair formation process, which requires localized linear actin polymerization (Mitchell et al, 1983; Lu & Adler, 2015), was severely impaired in the *pldo*-mutant cells.

**Figure S2.**
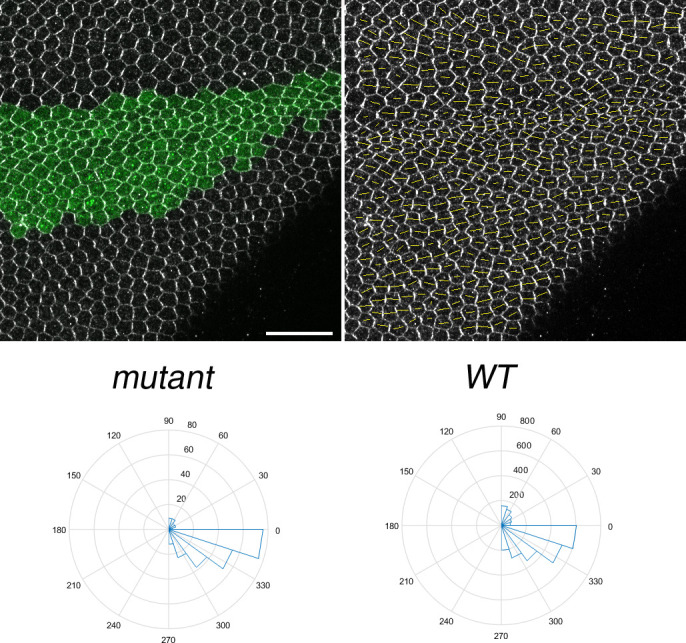
Pldo does not affect the core Fz/PCP pathway. Asymmetric Flamingo (Fmi) localization, as a proxy for Fz/PCP signaling, is shown. Note that it is not affected in *pldo*-mutant cells, as compared with surrounding control *wt* cells, in pupal wings (as shown in the graphs). Clones of mutant cells were generated by the MARCM technique, with mutant cells marked with GFP (green). Polarity strength lines of Fmi staining are shown as orange lines. Six different individuals were used, and around 100 cells were analyzed in each case. Scale bar represents 100 μm.

To further address the role of *pldo* in the hair formation process, we tested its relationship to the PCP pathway as Fz/PCP signaling is critical to focus actin polymerization to the distal vertex. Earlier stages of PCP establishment, preceding hair formation, are characterized by the asymmetric localization of its core components (e.g., Adler et al, 2013; Matis et al, 2014; and rev. in Strutt and Strutt [2009] and Carvajal-Gonzalez and Mlodzik [2014]). We thus evaluated if the asymmetric core PCP complex localization is affected. Fmi serves as the anchor of both distal and proximal PCP complexes, and its localization reflects this feature as a regular zig-zag pattern enriched in the proximal–distal axis, which can be visualized and quantified (Aigouy et al, 2010). We did not detect any changes in Fmi localization in wing cells mutant for *pldo*, as compared with neighboring control cells (Fig S2), indicating that Pldo is neither upstream nor part of the regulatory circuit of the core PCP pathway. This observation and interpretation are consistent with the phenotypes seen by altering the expression of core PCP factors or direct regulators of this pathway, with the actin hairs often being formed, but in abnormal number and/or locations (Yan et al, 2008; Lu et al, 2015).

A second feature regulated by the core PCP pathway, during pupal wing development and in general, is the organization of the epithelial cells themselves with their arrangement into a hexagonal pattern (Wong & Adler, 1993; Sánchez-Gutiérrez et al, 2013; Etournay et al, 2015). This terminal shape establishment follows a cell shape elongation along the proximo–distal axis, when PCP is reorganized from a radial polarity arrangement to a proximal–distal axis alignment (Aigouy et al, 2010). We thus asked whether *pldo* might be required in this context and compared the polygon distribution of *pldo*-mutant cells to surrounding wild-type control cells in pupal wing cells at 28–32 h APF. Interestingly, cells mutant for *pldo* display a reduction in the proportion of hexagonal shape, compared with surrounding control cells (Fig 2D), suggesting a role for Pldo in the cortical actin bundle function.

Taken together with the actin hair phenotype, these data suggest that Pldo likely acts as an effector of the core PCP pathway to regulate actin cytoskeleton–associated functions like cell shape definition and actin accumulation during hair formation and elongation. Consistent with this notion, *pldo* encodes protein domains associated with actin binding and regulation of its polymerization, such as WH2, FH1, and polyproline domains.

### Pldo promotes linear actin polymerization during hair outgrowth

**Figure S3.**
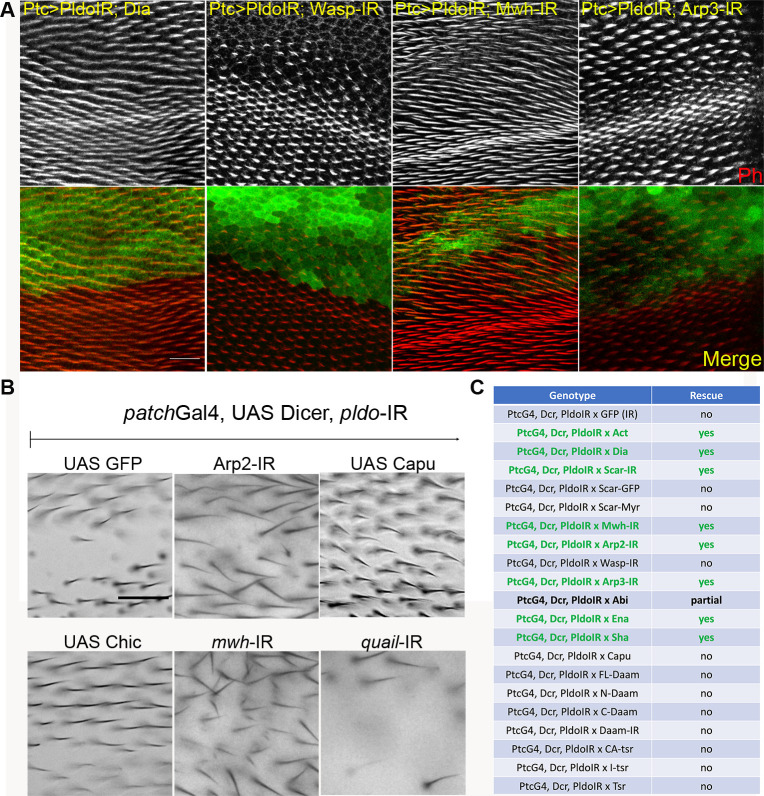
Pldo mediates competition for monomeric actin during trichome formation, extended. **(A)** *pldo* LOF phenotype reversion evaluated in pupal and adult wings. Confocal images of pupal wings expressing *ptcGal4*, *UAS-Dcr*, and *UAS-pldo*IR in combination with the indicated transgenes. Rhodamine conjugated to phalloidin was used to stain actin filaments, and GFP was used to mark the *ptcGAL4* domain in the wing. The scale bar corresponds to 50 μm. **(B)** Additional adult wings showing the interaction between *ptcGal4*, *UAS-Dcr*, and *UAS-pldo*IR and the indicated transgenes (see Fig 3C for quantification). Quantification was performed as described in the main text Fig 3C. The scale bar corresponds to 50 μm. **(C)** Table displaying all interactions assayed with *ptcGAL4*, *UAS-Dcr*, and *UAS-pldo*IR. Data are based on scoring at least 10 individuals in each case.

**Figure 3.**
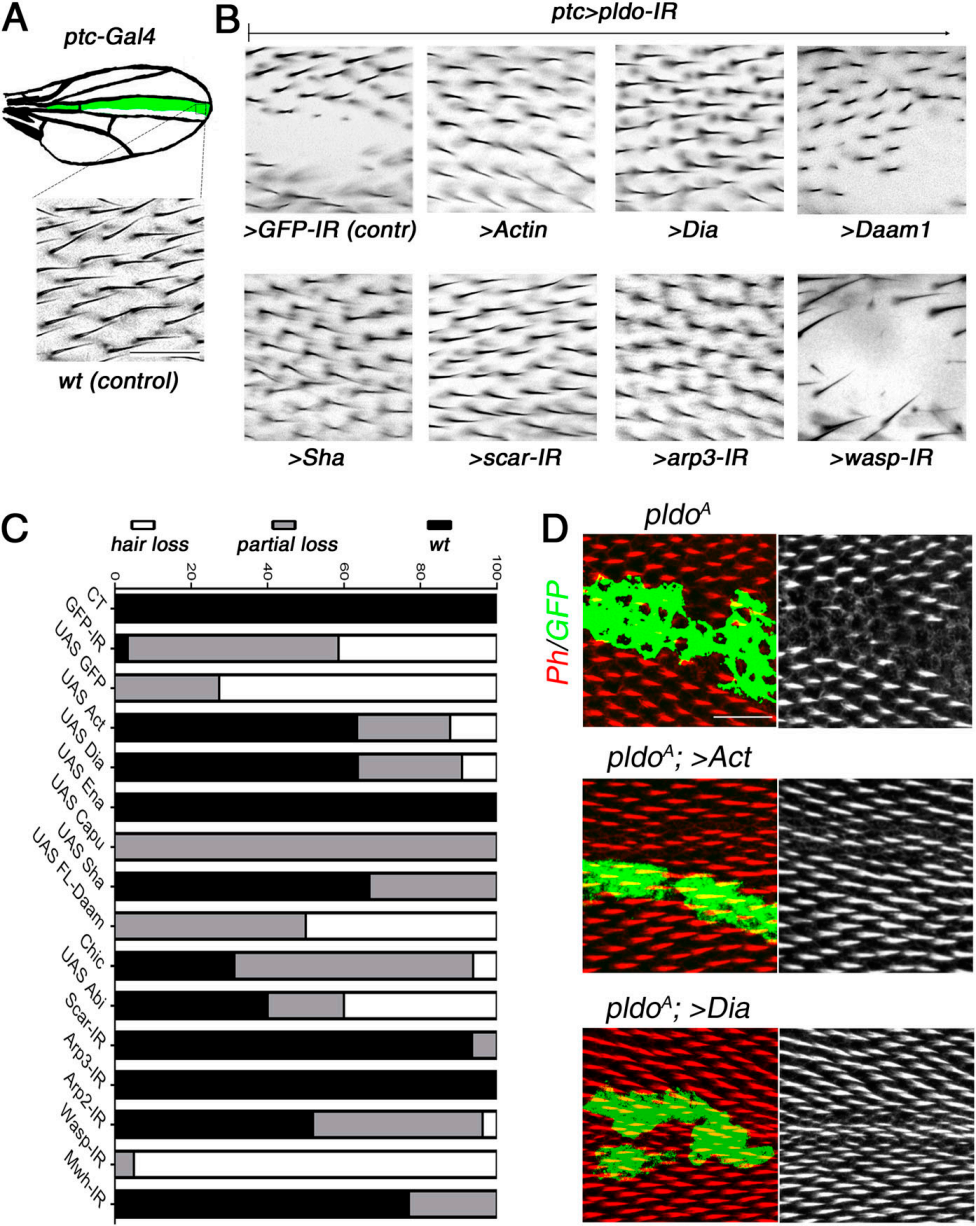
Pldo mediates competition for monomeric actin during trichome formation. **(A)** Schematic displaying the *ptc*Gal4 expression pattern (green area) used in pupal and adult wings. **(B)** Square in drawing corresponds to the region shown in the lower panel (*wt* control) and (B) panels. **(B)** Scale bar corresponds to 50 μm (also for panel B). **(B, C)** Genetic interactions between *pldo-IR* (knockdown) and factors that regulate actin cytoskeleton polymerization with phenotypic appearance as evaluated in adult wings. **(A)** Squares display high magnification views of wing area (boxed in schematic in A) expressing *ptcGal4*, *UAS-Dcr*, and *UAS-pldo-IR* in combination with the indicated transgenes, either knocking down additional factors or overexpressing a gene of interest. **(A, B)** Comparing the control phenotype of *ptcGal4*, *UAS-Dcr*, *UAS-pldo-IR*, and *UAS-GFP-IR* (upper left panel) to *wt* in (A) and the genotypes of interest (B). Potential reversion of the *pldo-IR* (LOF) phenotype was evaluated according to the size of the area in adult wings, which lost the trichomes. **(C)** Quantification was performed by scoring each wing with either “wild-type” (wt, normal hair number), “partial loss of trichomes” (partial loss), or “complete loss of trichomes” (hair loss) as shown in graph in (C). **(B, C)** Percentages of the respective appearance are shown in (C), see (B) for phenotypic reference: complete reversion to wild-type as seen with *UAS-scar-IR* and partial reversion displaying small areas with trichome loss as seen with *UAS-Capu* (Fig S3B) and no reversion (large area with no trichome) as seen in control *UAS-GFP-IR* or *UAS-wasp-IR*. Quantification is based on scoring >10 individuals in each case. **(D)** Reversion of the *pldo* (LOF) phenotype as seen in *pldo*-mutant MARCM clones in pupal wings to confirm the extent of phenotypic rescue. Note that actin and Dia overexpression/gain of function (GOF) showed a complete reversion of *pldo*-mutant clones in pupal wings. Scale bar corresponds to 25 μm.

To gain insight into the underlying regulatory mechanism(s) of how Pldo might function in actin filament dynamics, we asked whether the *pldo*-mutant phenotype can be genetically modified by genes encoding proteins regulating linear and/or branched actin polymerization. Such genes fall into the category of formins, including Dia or the anti-formin Mwh, or the WASP family, including Scar/WAVE and Wasp, among others (a list of actin regulators tested is shown in Fig S3C). Specifically, for example, Dia and Enabled/Ena promote linear actin polymerization, whereas Scar and Wasp induce branched actin polymerization by means of activating the Arp2/3 complex (Machesky et al, 1999; Kang et al, 2010; and rev. in Paunola et al [2002], Goley and Welch [2006], Le Clainche and Carlier [2008], Campellone and Welch [2010], Rotty et al [2012], and Carlier et al [2013]). Mwh, a functional anti-formin inhibits Dia function, preventing linear actin polymerization (Lu & Adler, 2015). To assay for potential interactions, we expressed *pldo-IR* under the control of *ptcGal4* (Fig 3A), causing a reduction of trichomes within the *ptc* expression domain and thus mimicking the *pldo* loss-of-function (LOF) phenotype. In this context, a phenotypic rescue of *pldo* was detected by increasing levels of proteins that induce linear actin polymerization, for example, Dia, and Ena in the *ptcGal4>pldo-IR* background (Figs 3B and C and S3C). Strikingly, the *pldo* LOF knockdown phenotype was also “rescued” by reducing Mwh levels (Figs 3C and S3A and B). Noteworthily, a *mwh* knockdown reversed the *pldo* LOF phenotype and produced the well-described “multiple wing hair” defects, characterized by the appearance of more than one short hair per cell (Fig S3A and B) (Yan et al, 2008; Lu et al, 2015). As mentioned, Shavenoid/Sha is another component required for trichome formation. *sha* LOF alleles show either the absence of hairs or the appearance of more than one tiny hair and thus has been described as an inducer of linear actin polymerization (Delon et al, 2003; Ren et al, 2006; Adler, 2018). We therefore tested whether increasing Sha protein levels allowed hairs to form in a *ptcGal4>pldo-IR* background. Indeed, Sha co-(over)expression in this background restored hair formation (Fig 3B and C). Interestingly, *pldo* LOF phenotypes in the antenna laterals (Fig S1E and F) are similar to the phenotype observed in *shavenoid* mutant flies (He & Adler, 2002). Taken together, our data indicate that promoting linear actin polymerization by increasing the levels of Dia, Ena, Sha, and Profilin/Chic in the *ptcGal4>pldo-IR* background restored hair formation and rescued the *pldo* LOF phenotype. A similar “rescue” was observed by reducing Mwh levels, an inhibitor of Dia activity.

Competition for actin monomers in the regulation of linear versus branched actin polymerization has been described (Jaiswal et al, 2013; Burke et al, 2014; Suarez et al, 2015; Vitriol et al, 2015; Davidson et al, 2018; and rev. in Davidson and Wood [2016] and Suarez and Kovar [2016]), and we thus asked whether a similar competitive nature exists during trichome formation. To address this, we asked whether reducing branched actin polymerization by knocking down *scar*, *arp2*, *arp3*, and *wasp* has an effect in the *ptcGal4>pldo-IR* background. Strikingly, we observed a reversion of the phenotype in most cases, including *scar*, *arp2*, and *arp3* knockdowns (Figs 3B and C and S3A and B). The exception was *wasp* knockdown (Fig 3B and C). However, as Scar and Wasp have specific roles in different cell types (Zallen et al, 2002; Berger et al, 2008; Rajan et al, 2009; Koch et al, 2012), these results suggest that Scar function is relevant in pupal wing cells, but Wasp is not required there. Taken together, these results suggest that competition for actin monomers during trichome formation in *Drosophila* is a critical component of its regulatory circuitry. To further corroborate this notion, we evaluated the effect of increasing actin monomer levels in the *ptcGal4>pldo-IR* background. Strikingly, a *ptcGal4>pldo-IR*, *>Act* genotype displayed a complete reversion of the trichome loss (Fig 3B and C), consistent with the hypothesis that Pldo influences the competition for actin monomers. Importantly, these phenotypic rescues were also observed when assayed in *pldo* MARCM clones and analyzed in pupal wings (see Fig 3D for examples).

In summary, these data suggest that the function of Pldo is to promote linear actin polymerization required during trichome formation, which could be either through the inhibition of branched actin polymerization or by directly inducing linear actin polymerization. The combination of either increasing actin itself and linear actin filament promoting factors or the reduction of branched filament promoting factors suggest that Pldo promotes linear actin polymerization by positively acting on its regulators and/or represses branched actin regulators. As such, Pldo functions in this context as a balance tipping factor toward linear actin polymerization, either by a direct promotion of linear actin polymerization or by inhibiting branched actin polymerization or both.

### Pelado is required for filopodia formation in hemocytes

**Figure 4.**
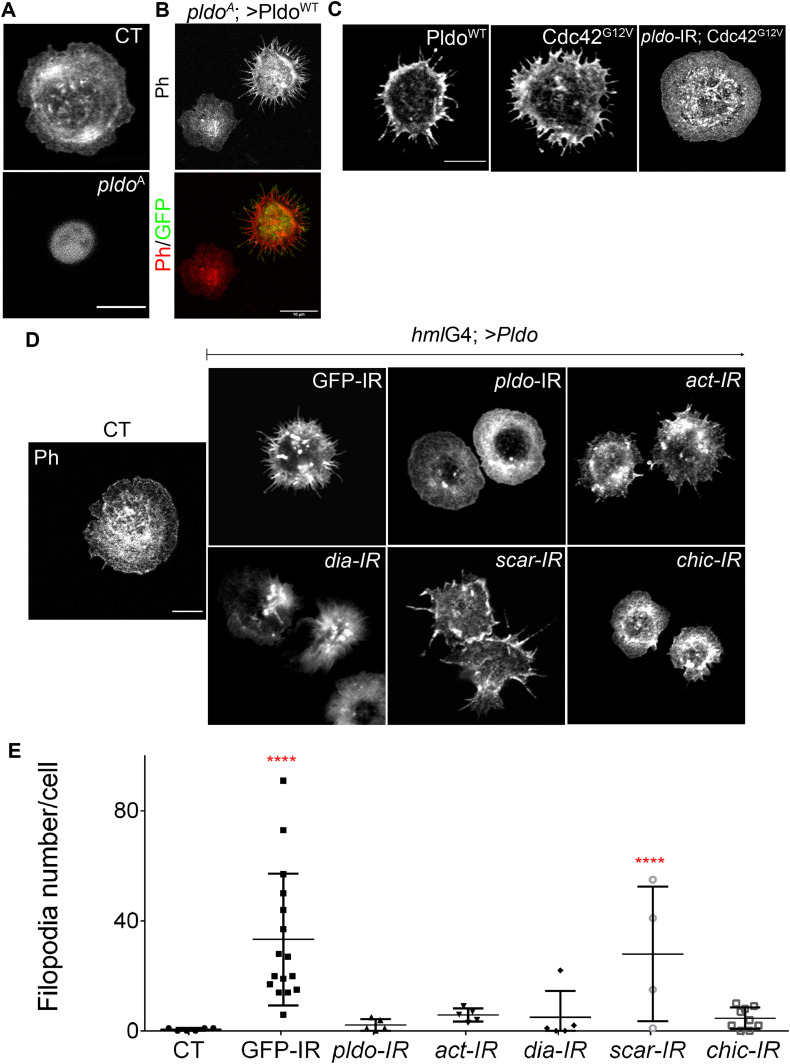
Pldo induces formation of filopodia in hemocytes. **(A)** Confocal microscopy images of hemocytes stained with rhodamine phalloidin to visualize actin filaments. Control wild-type hemocyte (CT, top) is shown in comparison to *pldo*-mutant cells, which show a reduction in the cell attachment area (*pldo*^A^, bottom panel). Scale bar = 5 μm. **(B)** Reduction in the cell attachment area was rescued by co-expression of Pldo^WT^. Note that such expression of Pldo^WT^ even creates a GOF phenotype, inducing the formation of filopodia-like protrusions. Rescued cell is marked by GFP (green) with the MARCM technique. Scale bar corresponds to 10 μm. **(C)** Pldo^WT^ expression in hemocytes induce filopodia formation, similar to Cdc42^G12V^ expression, a known inducer of filopodia. Formation of filopodia in Cdc42^G12V^ background depends on *pldo* function (also Fig S4D for quantification). Scale bar corresponds to 5 μm. **(D, E)** Filopodia formation induced by Pldo overexpression in hemocytes as driven by *hml-Gal4* driver (second panel from left, *GFP-IR* control, cf. to *wt* in the left most panel) was suppressed by knockdown (via IR) of either *pldo*, *act*, *Dia*, or *chic* (Profilin) and enhanced by *scar* knockdown. Scale bar corresponds to 5 μm. **(E)** Quantification of the number of filopodia per cell. At least 10 cells were quantified in each genotype in three independent experiments. Statistical analysis was performed by two-way ANOVA, Tukey posttest with ***P < 0.0001.

**Figure S4.**
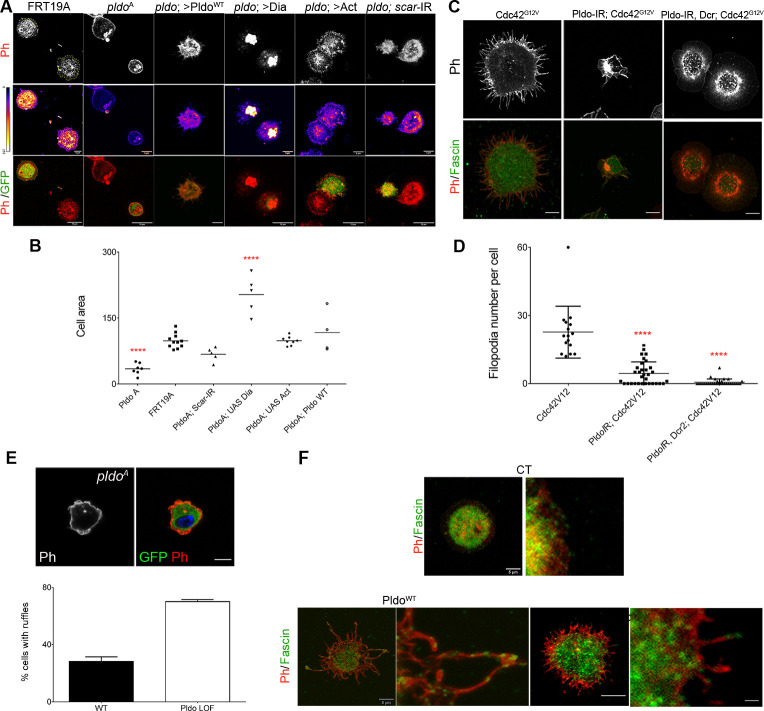
Pldo is essential to induce filopodia formation and to sustain cell shape in hemocytes. **(A)** *pldo*-mutant cells were generated by the MARCM technique (expressing GFP, green cells). An FRT19A chromosome was used as a wild-type control. Note that *pldo*-mutant cells show a reduction in the cell attachment area that was rescued by the expression of either Pldo, Dia, or Act or by knocking down *scar*. Scale bars correspond to 10 μm. **(B)** Cell area was quantified using the phalloidin staining (red and heatmap), using at least 10 cells per genotype in each experiment (and three independent experiments). Statistical analysis was one-way ANOVA, Tukey posttest *P* < 0.0001. **(C, D)** Filopodia formation induced by the expression of Cdc42^G12V^ in hemocytes, was evaluated by knocking down *pldo*. With or without Dicer expression in the LOF of Pldo, there was a significant reduction in filopodia formation by Cdc42^G12V^. Scale bars represents 5 μm. Number of filopodia per cell was quantified in at least 10 cells per genotype in each experiment (and three independent experiments). Statistical analysis was one-Way ANOVA, Tukey posttest *P* < 0.0001. **(E)** Ruffle formation in *pldo*-mutant cells. The ruffle presence was quantified in at least 10 cells per genotype in each experiment (three independent experiments). The scale bar represents 5 μm. **(F)** Fascin (green, staining bundled linear actin) serves as a marker for mature filopodia. Higher magnification of filopodia showing the fascin presence in the distal filopodial region in Pldo GOF or Cdc42G12V. The scale bar represents 5 and 20 μm in high magnification views.

Because the actin cytoskeleton is essential in every cell, and *pldo*-mutant animals die early in development, we assumed that Pldo is required in many if not all cells. The actin cytoskeleton is also important to regulate and maintain cell shape and changes in cell shape in *pldo*-mutant cells were observed in wing epithelial cells (Fig 2D). We thus next asked whether *pldo* is required to regulate cell morphology in hemocytes, a migratory *Drosophila* blood cell similar to mammalian macrophages (Moreira et al, 2013) (Fig 4A). *pldo*-mutant hemocytes (generated by the MARCM technique) displayed reduced attachment cell area and a significant increase in structures known as ruffles (Fig 4A, bottom panel, and Fig S4E). This phenotype was rescued by expressing Pldo^WT^ in this experimental setting (Fig 4B, rescued cell is marked by GFP). Exogenous expression of Pldo^WT^ in the *pldo*-mutant cells, likely an overexpression relative to its endogenous levels, did in fact not just rescue the LOF defects but caused an increase in the formation of filopodia-like protrusions (Fig 4B). This observation was consistent with its proposed role in favoring linear actin polymerization (which could be a direct effect or by preventing branched actin polymerization). Moreover, similar to the wing hair formation assay (Fig 3), the *pldo* hemocyte defects were also rescued by co-(over)expression of Dia, actin, or by IR-mediated LOF of *scar* (Fig S4A and B).

Pldo overexpression in hemocytes induces a significant increase in filopodia-like protrusions formation (Fig 4C, left panel). To confirm that these protrusions were filopodia, we stained the cells with anti-Fascin, a known marker of mature filopodia (Vignjevic et al, 2006). We observed Fascin staining in some of the filopodia, in all such conditions (Fig S4F). *cdc42* is a well-defined inducer of filopodia formation (Leung et al, 1998). As the Pldo-induced protrusions resembled the effect of expressing constitutively active Cdc42 (Fig 4C, middle and left panel, respectively), we thus asked whether Cdc42^G12V^-induced filopodia in hemocytes depend on Pldo function. Strikingly, an RNAi knockdown of *pldo* caused a total reversion of the Cdc42^G12V^ induced filopodia formation (Fig 4C, right panel, and Fig S4C and D). Together, these data indicate that Pldo is essential for filopodia formation in hemocytes downstream of Cdc42 and strongly supports the notion that Pldo directly favors linear actin polymerization.

Similar to the rescue experiments of trichome formation in pupal wings, Pldo-induced filopodia formation depends on regulators of linear actin polymerization, and as such, its filopodia-inducing effect was reversed by reducing the levels of Dia, Actin, or Chic/Profilin and enhanced by reduction in the *scar* gene, promoting branched actin (Fig 4D and E). Again, this is consistent with the notion that Pldo promotes linear actin polymerization in the cellular competition with actin monomers.

Taken together, the wing cuticle epithelial cell and hemocyte studies indicate that Pldo possesses a general function in regulating the competition for actin monomers and favoring linear actin polymerization over branched filaments.

### N-terminal region of Pldo is sufficient to induce filopodia formation

**Figure 5.**
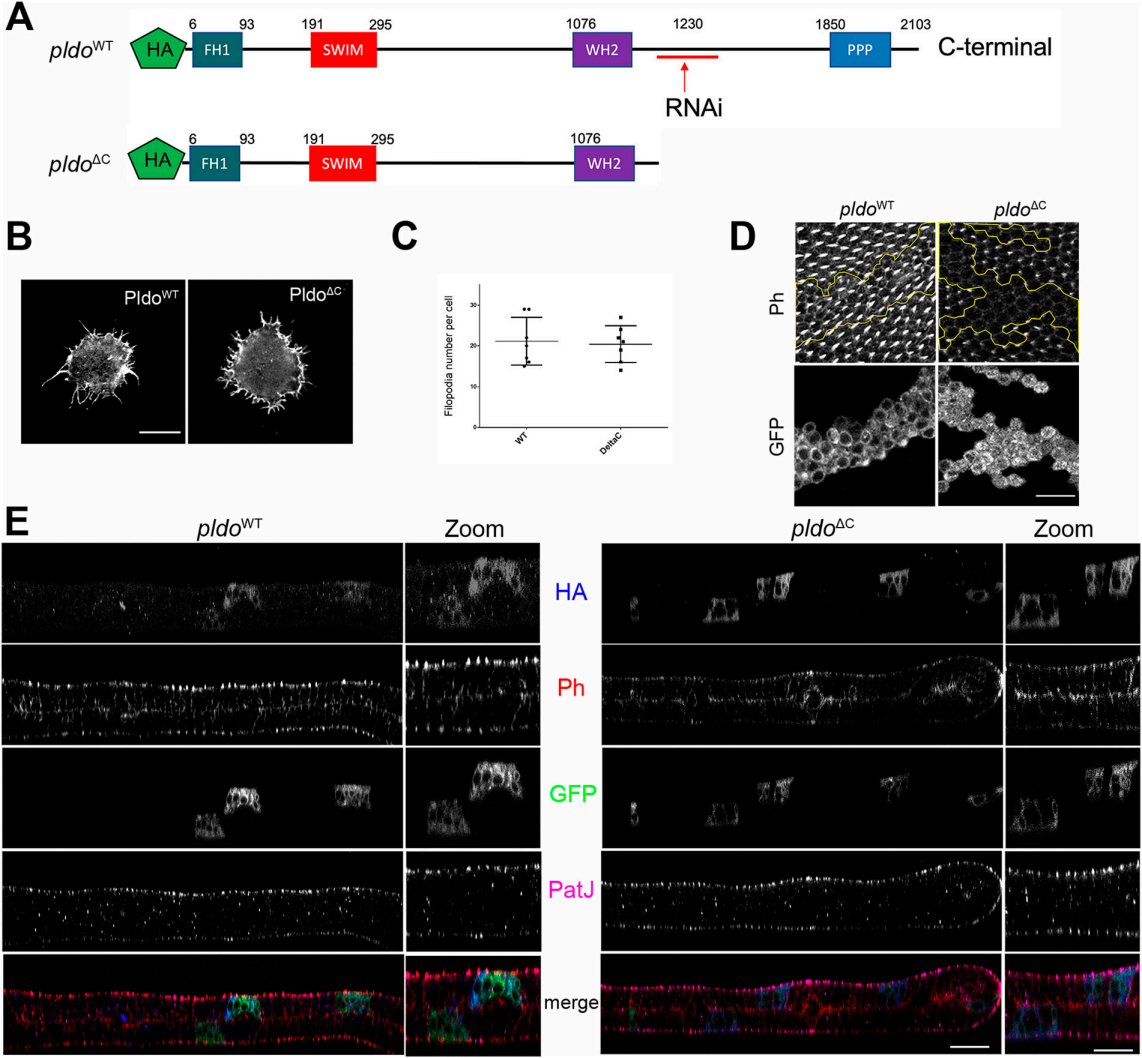
N-terminal region of Pldo is sufficient to induce filopodia formation. **(A)** Schematic of Pldo highlighting the deletion isoform with C-terminal deletion, Pldo^ΔC^ (note that the RNAi sequences are absent in the Pldo^ΔC^ construct). Both constructs have an HA tag at the N-terminus. **(B, C)** Pldo^ΔC^ was sufficient to induce formation of filopodia in hemocytes, to the same extent as full-length Pldo^WT^. **(B, C)** Examples shown in (B) and quantification in (C), showing the number of filopodia per cell. Scale bar represents 10 μm. At least 10 cells were analyzed in each condition in three independent experiments. Statistical analysis with *t* test, *P* = 0.8028, nonsignificant. **(D)** Expression of Pldo^ΔC^ in MARCM *pldo*-mutant cells (right panels) in pupal wings was not able to rescue hair formation, compared to *pldo*^WT^ expression (left panels). Distal is right. Scale bar represents 25 μm. **(E)** Subcellular localization (z-plane) analyses of HA-tagged Pldo^WT^ (left panels) and Pldo^ΔC^ (right panels), displaying rhodamin phalloidin (Ph, red) staining actin filaments, clonal marker (GFP, green, via MARCM, mutant cells), PatJ (magenta) as a junctional (apical) cell marker, and HA-Pldo (blue) showing Pldo localization. Note that there is no detectable difference in the localization of Pldo^WT^ and Pldo^ΔC^ (HA staining, blue) in pupal wing cells. Right panels (zoom) show higher magnification. Scale bar represents 25 μm.

**Figure S5.**
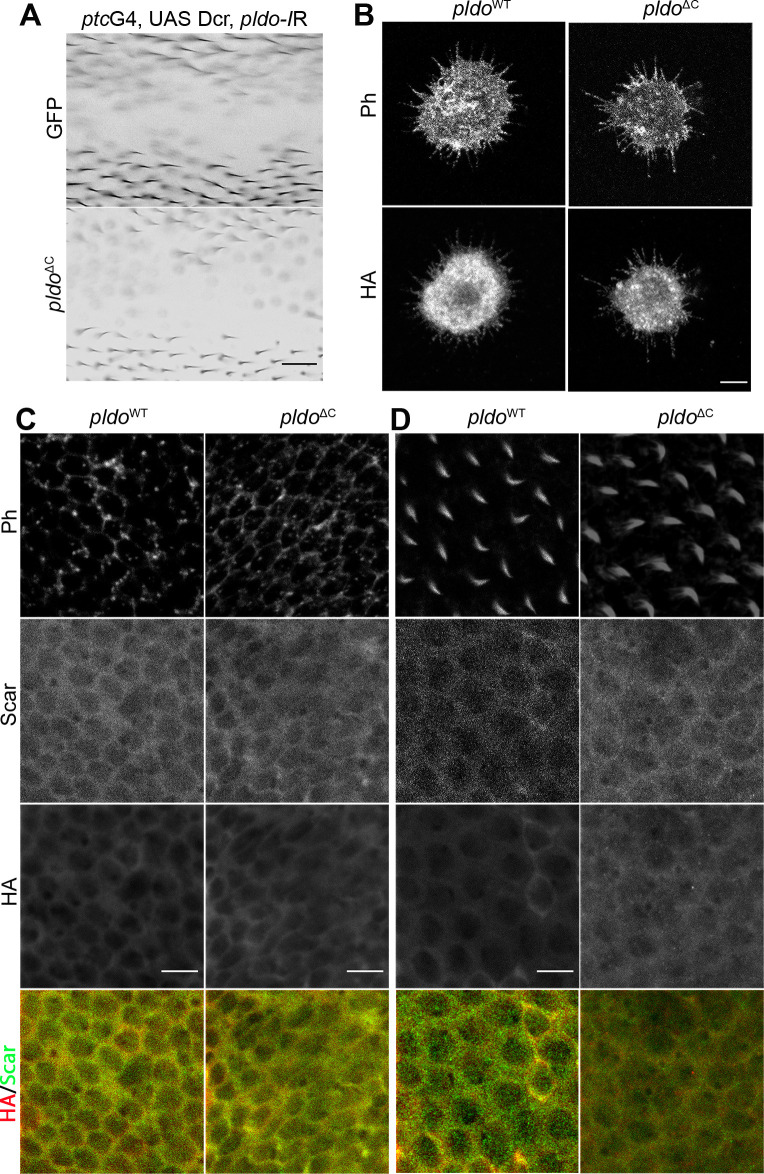
N-terminal region of Pldo is sufficient to induce filopodia formation. **(A)** Pldo^ΔC^ was not capable to rescue the lack of trichome phenotype observed by *pldo* LOF in adult wings, when co-expressing with the *UAS-pldo-*IR in the *ptcGAL4* expression domain. No change in the phenotype was observed, confirming the *pldo* LOF mutant defects, as seen in pupal wings (Fig 5D). The scale bar represents 50 μm. **(B)** Localization of Pldo was evaluated in confocal microscopy images, through the HA-tag staining, in hemocytes, observing no notable differences. These experiments were performed at 18°C, with near wild-type expression levels to prevent overexpression of the transgenes. Scale bar represents 5 μm. **(C, D)** Localization of Pldo was evaluated in confocal microscopy images, through the HA-tag staining, in pupal wings, before and after hair formation, observing no notable differences. **(C, D)** These experiments were performed at 18°C, at 64 (C), or 72 h (D) APF, with near wild-type expression levels to prevent overexpression of the transgenes. Note at the bottom the colocalization between Pldo-HA and Scar. The scale bar represents 25 μm.

To evaluate which regions of the Pldo protein were essential for its function in actin polymerization, we generated a mutant isoform deleting its C-terminal portion (Pldo^ΔC^) (Fig 5A). We first compared the gain-of-function (GOF) phenotypes of Pldo^WT^ and Pldo^ΔC^ in the hemocyte assay, noting that both Pldo^*WT*^ and Pldo^ΔC^ induce filopodia formation equally well (Figs 5B and C and S5B).

In the context of actin hair formation in wing cells, however, Pldo^*WT*^ fully rescued the *pldo* LOF mutant phenotype, but expression of Pldo^ΔC^ in mutant clones did not rescue the loss-of-hair defect (Fig 5D). Similarly, Pldo^ΔC^ expression did not rescue the loss-of-hair phenotype in the *ptcGal4>pldo-IR* background (Fig S5A). These results indicate that the N-terminal portion of Pldo is sufficient to induce linear actin polymerization, for example, in hemocytes, but that its C-terminal region is required in more complex situations in epithelial cells, like trichome formation, likely because of a regulatory function or for subcellular localization. To test for the latter, we asked whether the C-terminal portion of Pldo is required to localize the protein to a specific region within the wing cells. Surprisingly, we did not detect a difference in the localization between Pldo^WT^ and Pldo^ΔC^ in either wing cells (Figs 5E and S5C and D), or hemocytes (Fig S5B). Similar to Scar (Zallen et al, 2002; Kunda et al, 2003; Beli et al, 2008; Rodriguez-Mesa et al, 2012), Pldo localization is detected throughout the cell, which has also been noted for its human ortholog ZSWIM8 (Okumura et al, 2021).

In this context, we also tried to identify the minimal portion of Pldo required to induce linear actin polymerization by generating additional C-terminal deletions (Pldo^1-685^ and Pldo^1-515^ isoforms, as compared with 1–1,105 on Pldo^ΔC^). However, these constructs were cell lethal, in both *Drosophila* S2 cells and human A549 cells.

### Pldo function in actin cytoskeletal regulation is conserved in mammalian cells

We next wished to determine whether the function of Pldo in actin regulation is a conserved feature of the protein and used a cell migration assay in human cells to this end. Cell migration is important in many different developmental and physiological processes and requires specific actin cytoskeletal dynamics in a highly regulated process (rev. in Le Clainche and Carlier [2008]). Different cells use distinct mechanisms for migration and can also change between different types of migration. The common component in the distinct mechanisms of cell migration is actin. Similarly, a key feature during cell migration is the up-regulation of the Arp2/3 complex activity (rev. in Swaney and Li [2016], Jockusch [2017], and Shellard and Mayor [2020]). We thus used a cell migration assay as a tool for an initial insight whether Pldo function in actin regulation is conserved in human cells.

**Figure 6.**
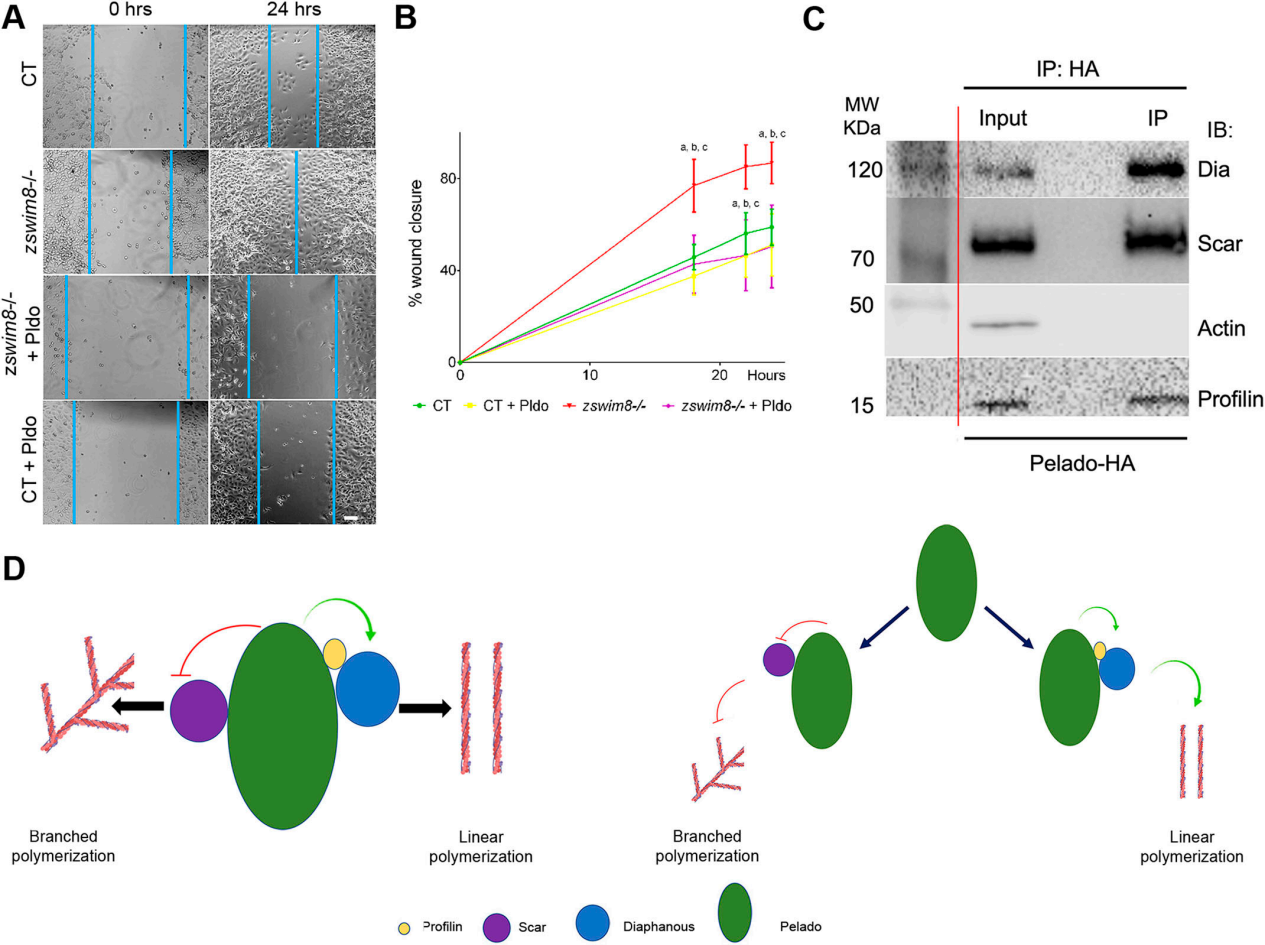
Pldo function in actin cytoskeletal regulation is conserved in human cells. **(A, B)** Pldo/ZSWIM8 function was evaluated in a scratch wound migration assay in human cells (cell line A549). *pldo/ZSWIM8* mutant cells showed a significant increase in the rate of migration during assay. This effect was reversed by expressing the *Drosophila* *pldo* gene (independent experiments: n = 3). Scale bar represents 50 μm. **(B)** Quantification of % wound closure over time. Statistical analysis was performed via a two-way ANOVA, Tukey posttest, *P* < 0.0001, “a” indicates ** between CT and *zswim8−/−*; “b” indicates *** between CT+Pldo and *zswim8−/−*; and “c” indicates *** between *zswim8−/−* and *zswim8−/−* +Pldo. **(C)** Immunoprecipitation (IP) assay documenting an interaction of Pldo with different proteins that regulate actin cytoskeletal dynamics in *Drosophila* S2 cells transfected with HA-Pldo^WT^ and immunoprecipitated with anti-HA. Note that Dia, Scar, and Chic/Profilin all co-IP with Pldo, suggesting that they form a complex. Actin was not co-IPed with Pldo. The vertical red line indicates a blot crop bringing the molecular weight markers in proximity of the IP-ed proteins. **(D)** Schematic of proposed models for Pldo function: either forming a multiprotein complex with Scar, Dia, and Chic/Profilin during the regulation of actin dynamics (left side) or binding to these proteins independently (right side). Source data are available for this figure.

To this end, we used the human A549 cell line (kindly provided by the Bartel laboratory [Shi et al, 2020]). We established a clonal knockout cell line for *ZSWIM8*^*−/−*^, with *ZSWIM8* being the human *pldo* ortholog, and analyzed its effect in a cell migration wound healing assay. Strikingly, cells mutant for *pldo/ZSWIM8* showed an increase in the migration rate, as compared with wild-type control A549 cells (Fig 6A and B). Importantly, this effect was fully reversed by the expression of *Drosophila* Pldo in those cells (Fig 6A and B), confirming that ZSWIM8/Pldo also regulates actin polymerization in mammalian cells and that the *Drosophila* Pldo protein is functionally equivalent in this context. These data are very similar to experiments demonstrating that Arp2/3 complex activity and, as consequence, branched actin filament formation are essential in the wound healing assay (Zhao et al, 2020; using the same cell line) and thus suggest that Pldo’s function is also to prevent branched actin polymerization.

Taken together, our data indicate that the Pldo/ZSWIM8 function in the regulation of actin cytoskeletal elements is conserved between *Drosophila* and human cells and further confirm the notion that these proteins function in general to favor linear actin polymerization over branched filaments.

### Pldo physically associates with regulators of actin polymerization

The actin polymerization regulators mDia2 and Scar/WAVE are known to be part of the same molecular complex and that the binding of Cdc42 modulates their interaction to induce filopodia formation (Krugmann et al, 2001; Beli et al, 2008; Goh et al, 2012). We thus asked whether Pldo is also associated with these and/or other proteins that regulate actin cytoskeleton dynamics. To test this hypothesis, we used the *Drosophila* S2 R+ cell line (Schneider, 1972; which is a macrophage-like lineage derived from late embryos), and we observed that Pldo was indeed part of a molecular complex with Dia, Scar/WAVE, and Chic/Profilin (Fig 6C). We also evaluated the potential colocalization of Pldo and Scar in pupal wing cells. These data are however not informative as both proteins are detected throughout the whole cell (Fig S5C). Together, these observations suggest that Pldo could be regulating the activity of these proteins as part of the described complex (Miki et al, 2000; Krugmann et al, 2001; Beli et al, 2008; Goh et al, 2012), including Dia and Scar/WAVE.

**Figure S6.**
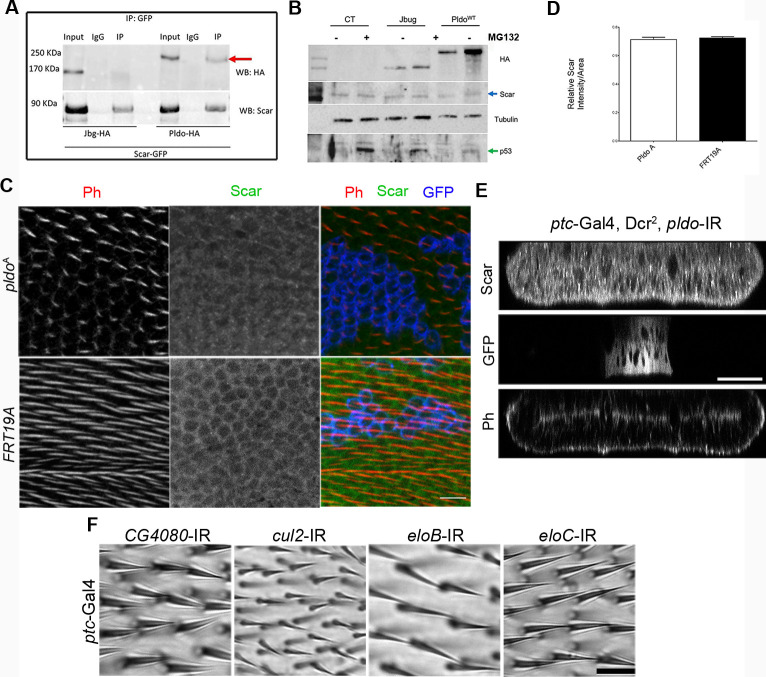
Pldo function in actin cytoskeleton regulation is not related to the ubiquitin ligase complex activity. **(A)** Two independent publications have shown that the Pldo homologs EBAX-1/ZSWIM8 form part of a ubiquitin ligase complex serving an E3 ligase function, recognizing the substrate targets to be degraded by the proteasome. We did not observe such a role of the gene in the actin dynamics context. IP assay evaluating the interaction of Pldo and Scar. S2 cells were co-transfected with Pldo-HA and Scar-GFP and were immunoprecipitated with anti-GFP. Jitterbug-HA was used as a control (Jbug-HA). Note Scar immunoprecipitated with Pldo^WT^, suggesting that they form a complex. **(B)** Scar levels were evaluated in S2 cell protein extracts by Western blot in the presence or absence of the proteosomal inhibitor MG132. Scar levels were compared between untreated cells, Jbug expressing cells as control, and Pldo expressing cells, each of them with and without MG132. No significant difference was observed in either case. Levels of p53 were used as a positive control. **(C, D, E)** Scar levels were tested in vivo as a potential target of the ubiquitin ligase complex, but no detectable differences were observed. FRT19 clones of wild-type were generated as a control (equivalent to wild-type tissue). **(D)** Quantification is shown in (D): At least three individuals were considered in each experiment and each condition: independent experiments n = 3. Statistical analysis was done via a *t* test. Scale bars correspond to 25 μm. **(E)** Scar levels and localization were also analyzed in Z-sections in pupal wing cells, in the *ptc*-Gal4 > *pldo*-IR background, using confocal Z-line tool; the *pldo* knockdown region is marked with GFP. No observable differences in Scar levels/localization between the *pldo* LOF region and the rest of the wing were detected. Experiments were performed at 25°C and 29°C. Scale bar represents 50 μm. **(F)** The effect of other components of the ubiquitin ligase complex was evaluated in adult wings, using the *ptc*-Gal4 driver to express RNAi transgenes of the different components of the ubiquitin ligase complex (as linked to EBAX in in *Caenorhabditis elegans*). Two different RNAi constructs were used for EloB, EloC, and Cul2, and all of them tested at 25°C and 29°C. None showed altered hair formation. Scale bar represents 25 μm. Source data are available for this figure.

Two reports characterizing EBAX-1, the *pldo* ortholog in *C. elegans*, and human ZSWIM8, respectively (Wang et al, 2013; Shi et al, 2020), suggest that it can function as an E3 ubiquitin ligase, being part of a ubiquitin ligase complex including elongins B and C, and Cullin-2 (Wang et al, 2013). Because *pldo* LOF is reversed by reducing Scar levels and Pldo and Scar are mutually co-immunoprecipitated (Fig 6C, also Fig S6A) and colocalized in pupal wings (Fig S5C), we hypothesized that Scar could be a target of such a Pldo function. Accordingly, if Scar were the target of Pldo in the context of a ubiquitin ligase complex, we would expect a change in Scar levels in mutant cells for *pldo*. However, we did not observe an effect of *pldo* on Scar protein levels or localization, as compared with wild-type control cells (Fig S6C–E). To further test this hypothesis, we evaluated Scar levels in S2 cells with or without the proteasome inhibitor MG132. If Pldo was promoting Scar degradation, we would expect an increase in Scar levels upon MG132 treatment. However, MG132 did not cause an accumulation of Scar (Fig S6B), whereas p53 (as a control protein known to be degraded by the proteasome) (Scheffner et al, 1993; Maki et al, 1996; Haupt et al, 1997; Honda et al, 1997; Ashcroft & Vousden, 1999; Yang et al, 2017; and rev. in Pant and Lozano [2014]) was increased. To address this further in vivo during trichome formation, we tested whether the knockdown of components of the ubiquitin ligase complex (described to act with EBAX-1 in *C. elegans*) would have an effect on hair formation (similar to the *pldo* LOF phenotype). However, LOF knockdown of elongins B/C or Cullin2 showed no defects in hair formation (Fig S6F). The same was observed for the knockdown of *CG4080*, a described E2 ligase that might act with Pldo (Fig S6F). These results indicate that the ubiquitin ligase activity is not required for linear actin polymerization during trichome/hair formation in *Drosophila*.

In summary, our data are consistent with a model where Pldo regulates actin dynamics inducing linear actin polymerization, either by promoting delivery of actin monomers toward linear actin polymerization or by promoting Dia function and/or inhibiting Scar/WAVE. Pldo might be functioning as part of a multiprotein complex, where it regulates both proteins; alternatively, it may interact independently with either Dia or Scar, depending on the cellular requirements (see model in Fig 6D). Pldo could be thus acting as a molecular switch to regulate actin polymerization, either toward branched or linear filaments, in different cell contexts and/or by binding to distinct actin regulators (see the Discussion section).

## Discussion

Here, we describe a lethal recessive mutation in the *pelado* (*pldo*) gene that, when analyzed in mosaic animals, results in external body regions lacking actin-based (hair) structures. Functional analyses of *Drosophila* Pelado (Pldo, also known as ZSWIM8 in humans and other species) in the context of the regulation of actin polymerization dynamics indicate that it is an essential component required for linear actin polymerization. Our studies support a conserved function of Pldo/ZSWIM8 in promoting linear actin polymerization—at the expense of or in competition of branched polymerization—in several cell types, ranging from *Drosophila* epithelial cells to a human cell line. Pldo/ZSWIM8 proteins share multiple domains that have been implicated in the regulation of actin filament dynamics, and Pldo/ZSWIM8 physically interacts with actin polymerization regulators, including Scar/WAVE, the formin Diaphanous, and Profilin. Functional dissection reveals specific domains of Pldo/ZSWIM8 being required for filopodia formation and other protein regions functioning in specific cellular contexts. Taken together, our data indicate that the N-terminal portion of Pldo/ZSWIM8 has a conserved regulatory function that is critical for linear actin polymerization. Although ZSWIM8 has been linked to ubiquitination in some contexts (Wang et al, 2013; Shi et al, 2020), our data argue against such a requirement during the regulation of actin dynamics.

### Pldo/ZSWIM8 is required for linear actin polymerization

During development and morphogenesis, *Drosophila* cuticular epithelial cells differentiate to produce the adult phenotypic adaptations as part of the exoskeleton. Wing development, leading to largely hexagonal cells with each cell producing an actin-based hair, serves as an excellent model to study the role of cellular morphogenesis and the regulation of cytoskeletal dynamics (Lu & Adler, 2015). The actin-based cellular hair, or trichome, covers all wing cells and most parts of the external body in *Drosophila* with the function of the trichome presumably related to a passive role, directing airflow over the body and wing (Guild et al, 2005).

The positioning of the actin hairs, in the distal vertex of each cell, depends on the core Fz/PCP pathway and its effectors, which control the accumulation and polymerization of actin fibers at the apico-distal region of every cell (rev. in Strutt and Strutt [2009], Adler [2012], and Carvajal-Gonzalez and Mlodzik [2014]). Seminal work revealed that hair formation is highly dependent on linear actin polymerization (Mitchell et al, 1983). Hair outgrowth in the wing epithelia has been divided in three different stages: at 28 h APF, actin starts to accumulate in the apico-distal zone of wing cells, aided by tubulin-dependent transport and vesicle recycling (Gault et al, 2012), directed by the activity of the core PCP pathway; at 30–32 h APF, the prehair structure becomes apparent corresponding to the early stages of hair growth, which is focused in one distal spot because of the inhibitory activity of Mwh (rev. in Adler [2012] and Carvajal-Gonzalez and Mlodzik [2014]). Subsequently, at 32–36 h APF, the hairs extend and are completely formed (at 36 h APF), mainly by elongation of linear actin filaments, arising from the “prehair” foci. A detailed analysis of *pldo/zswim8* mutants during the different stages of hair growth suggests that it is required for the formation of the prehair and linear actin extension within the hair. In addition, *pldo/zswim8* appears to affect cell shape morphogenesis with a reduction in hexagonal cell shapes, consistent with a role in linear cortical actin filaments. The observation that overexpression of the formin Dia can rescue the loss-of-function (LOF) *pldo/zswim8* phenotype resulting in loss of hairs, supports the notion that Pldo/ZSWIM8 regulates formin-dependent linear actin polymerization. The other described genes whose LOF generates the absence of cellular hairs in *Drosophila* are *shavenbaby* (*svb*) and *shavenoid* (*sha*). Svb is a transcription factor that induces the expression of Sha, which has been defined as a protein that regulates the actin cytoskeleton. However, *sha* is not conserved in the animal kingdom, and its presence is specific to insects and arthropods (He & Adler, 2002; www.flybase.org).

Actin hair formation, both number and location, is regulated by the core Fz/PCP pathway, but little is known about the mechanisms that regulate the actual actin polymerization during this process. Core PCP factor polarization, as exemplified by Fmi, is not affected in *pldo/zswim8* mutant cells, suggesting that Pldo/ZSWIM8 is a downstream effector of the PCP pathway. Dsh accumulates at the apico-distal zone of *Drosophila* wing cells, where it is thought to recruit and activate RhoA, Rac, and Daam1 (disheveled-associated activator of morphogenesis 1, originally identified in *Xenopus*) (Habas et al, 2001). Daam1 and likely also other formins, like Dia (as Daam1 appears to be redundant in this wing hair context), interact with Profilin to induce actin polymerization (Matusek et al, 2008; Barko et al, 2010; Yaniv et al, 2020; and rev. in Goodrich and Strutt [2011], Adler [2012], and Singh and Mlodzik [2012]). The *pldo/zswim8* LOF phenotype is rescued by Dia or Sha overexpression, whereas different isoforms of dDaam1, including activated ones (Matusek et al, 2006), do not rescue *pldo/zswim8* LOF defects, consistent with the notion that Dia and not Daam1 is the critical formin family member in this context, in analogy to Scar and Wasp with each acting in a cell type–specific manner or process. A direct link of Dia and the Fz-Dsh complex has not yet been reported, and thus, the connection between Pldo/ZSWIM8 and core PCP complexes remains unclear. Further work will be required to establish a better understanding of the information flow from core PCP factors to the regulatory interactions at the actin polymerization level.

Besides the actin hair/trichome, *pldo/zswim8*–mutant epithelial cells also display cell shape defects, which—based on its link to actin—are likely caused by defects in cortical actin regulation. As the cortical actin cytoskeleton fulfills an essential role in all cells, it is consistent that a *pldo/zswim8* mutant is embryonic lethal, albeit with very variable phenotypes. It should be noted that the highest levels of *pldo/zswim8* message are maternal in the early embryo, but germline clones of *pldo/zswim8* do not survive to allow egg development and deposition, likely because of defects during oogenesis.

### Pldo/ZSWIM8 affects the balance of linear versus branched actin filament polymerization

Loss of the actin hair in *pldo/zswim8–* mutant cells is not only rescued by the overexpression of the formin Dia and actin itself but also by the knockdown of *scar* and *arp2/3*. These “synthetic” genetic interactions suggest that Pldo/ZSWIM8 affects the balance of linear versus branched actin polymerization, with either an excess of actin monomers or the reduction of the capability of the cell to promote branched actin, changing the balance back to linear actin. Thus, Pldo/ZSWIM8 facilitates the process by favoring linear polymerization. Notably, *wasp* knockdown did not reverse the *pldo/zswim8* mutant phenotype, as compared with *scar*, which efficiently reversed the *pldo/zswim8* defect. It has been described that Scar and Wasp act independently, regulating branched actin polymerization in different developmental contexts, as for example, Scar is important for morphogenetic cell processes, whereas Wasp being essential during muscle and sensory organ development (Brüser & Bogdan, 2017). Interestingly, a knockdown of *mwh*, an anti-formin inhibiting Dia, allowed also a partial phenotypic reversion of the *pldo/zswim8* defect, suggesting that Dia function is rate-limiting in *pldo/zswim8* mutants (*mwh* is epistatic to *pldo/ZSWIM8* as the *mwh* LOF hair phenotype is manifested in the double knockdown, with 3+ hairs forming per cell; Yan et al, 2008).

Although cells generally have a high concentration of actin monomers (Koestler et al, 2009; Burke et al, 2014; and rev. in Pollitt and Insall [2009]), most of it is sequestered by different regulatory proteins, like Profilin and Thymosinβ4, establishing a competition for actin monomers between the two distinct forms of polymerization (Burke et al, 2014; Henson et al, 2015; Suarez et al, 2015; Suarez & Kovar, 2016). With most of actin polymerization tilted toward branched, linear actin polymerization is more regulated than branched filaments.

The notion that Pldo/ZSWIM8 favors linear actin polymerization is also supported by the phenotype observed in hemocytes. Here, *pldo/zswim8*–mutant and Pldo/ZSWIM8 overexpression backgrounds generate cell morphology changes involving rearrangements in actin-dependent structures, like filopodia and lamellipodia. *Pldo/zswim8*–mutant cells display a rounded morphology with less surface contacts and increased ruffles, structures mainly based on branched actin polymerization (Legg et al, 2007; and rev. in Campellone and Welch [2010]). Similar observations were made for Scar gain-of-function (GOF) scenarios in BG3 cells from *Drosophila* (Cloud et al, 2019). It is noteworthy that cell adhesion to the matrix is based in focal adhesion–related structures, with focal adhesions directly interacting with the substrate and being associated with linear actin filaments, known as stress fibers (rev. in Jockusch [2017] and Hohmann and Dehghani [2019]). [These observations in hemocytes reinforce the idea that Pldo/ZSWIM8 functions to promote linear actin polymerization. Accordingly, Pldo/ZSWIM8 overexpression in hemocytes induces the formation of filopodia, structures essentially formed by long linear actin filaments. Importantly, Pldo/ZSWIM8 LOF in hemocytes expressing the constitutive active form of Cdc42, prevented filopodia formation, strongly suggesting that Pldo/SWIM8 has a direct role in inducing linear actin polymerization. To further address this, we looked for potential binding sites of Pldo to Cdc42 or other small GTPases (through the CRIB domain), but we did not detect such domains in the Pldo sequence. This could indicate that Pldo is not a direct target of Cdc42 or other small GTPases but that it functions as a downstream effector of a direct target of these GTPases.

Taken together, Pldo/ZSWIM8 serves a “balance” function promoting linear actin polymerization, probably via Dia, as a formin, inducing linear actin polymerization, in competition with Scar, inducing branched actin polymerization. Based on our data, the balance between linear and branched actin polymerization appears to be a critical determinant in the trichome formation process. The effect of Pldo/ZSWIM8 may be comparable to the one described for Profilin (Suarez et al, 2015), which favors linear actin polymerization over branched filaments in fission yeast cells.

### Pldo/ZSWIM8 is required for filopodia and related structures formation

The actin hair outgrowth process in *Drosophila* epithelial cells is similar to filopodia formation, with both being protrusions of the plasma membrane driven by linear actin filament extension. Similar to the epithelial actin hair context, *pldo/zswim8*–mutant defects in hemocytes are reversed by enhancing linear actin polymerization either by (i) increasing formin activity by overexpressing Dia, (ii) reducing branched polymerization by knocking down Scar, or (iii) by increasing actin monomer availability via overexpressing actin itself.

Importantly, this function of Pldo/ZSWIM8 is conserved from *Drosophila* to mammals. In a well-established wound healing cell migration assay in the human A549 cell line (Piao et al, 2014; Han et al, 2016; Wang et al, 2017; de Oliveira Rodrigues et al, 2020; Kim et al, 2020; Liao & Peng, 2020; Zhao et al, 2020), we observed that *pldo/zswim8* knockout cells migrate significantly faster than control cells, suggesting that these cells have an increase in branched actin polymerization, allowing a faster migration. Strikingly, this effect is rescued by providing the *Drosophila* Pldo/ZSWIM8 protein to these knockout human A549 cells, which would increase the competition for actin monomers, reducing the branched polymerization. This not only suggests a conserved function but also confirms that the *Drosophila* factor is functionally equivalent to the human protein. Very similar results were described by knocking down WDR63, an inhibitor of the Arp2/3 complex, causing an increase in branched polymerization and cell migration in A549 cells (Zhao et al, 2020), opposite to the effect observed when *arp2* is knocked down. These results are fully consistent with our data in *Drosophila* contexts, indicating that Pldo/ZSWIM8 changes the balance between linear and branched actin polymerization. This might be mediated possibly by either reducing Scar/WAVE activity (and in consequence Arp2/3–dependent branched actin nucleation) or by promoting Dia/formin function and hence linear actin polymerization, or both.

In summary, using multiple different cell types and experimental strategies, we have demonstrated that Pldo/ZSWIM8 serves a conserved function to promote linear actin polymerization at the expense of branched actin. Our results are all consistent with the notion that Pldo/ZSWIM8 functions in the same direction as Dia and Profilin/Chic activity and opposed to Scar and Arp2/3 activities in promoting linear actin polymerization and filopodia-like structures. Pldo/ZSWIM8 favors actin monomer competition and recruitment toward linear filament polymerization. Altogether, our results suggest that Pldo/ZSWIM8 is able to induce linear actin polymerization, as is required for filopodia formation in hemocytes, at the expense of branched actin polymerization (as shown in *pldo/zswim8* LOF experiments in hemocytes and human cells).

### Pldo/ZSWIM8 as a part of a molecular complex

Besides the predicted domains observed in the Pldo sequence (Fig 1D), there are also several tyrosine-binding motifs to SH2 (Src homology domain 2) and proline-rich SH3 domains detected in its sequence. In addition, a couple of binding motifs for EVH1 domain (enabled/vasodilator-stimulated phosphoprotein homology 1) are present.

mDia2, Arp2/3, and Scar/WAVE can form a multimolecular complex, and these interactions are modulated by Cdc42 (Miki et al, 2000; Krugmann et al, 2001; Beli et al, 2008). We tested for co-immunoprecipitation of Pldo/ZSWIM8 with different proteins that regulate actin filament dynamics. Pldo/ZSWIM8 appears to be part of a complex with Dia, Scar, and Profilin, suggesting that Pldo/ZSWIM8 is part of a related molecular complex to the one described (Miki et al, 2000; Krugmann et al, 2001; Beli et al, 2008). Accordingly, Pldo could be interacting with the Enabled (Ena) protein through its EVH1 domain (Ball et al, 2000; Hwang et al, 2021). IRSp53, which contains SH3 domains, has been described to interact with cytoskeletal regulatory proteins, like Scar/WAVE, Dia, and Mena (Miki et al, 2000; Jung et al, 2001; Krugmann et al, 2001; Suetsugu et al, 2006; Abou-Kheir et al, 2008; Goh et al, 2012; Stark et al, 2017; Pipathsouk et al, 2021). Thus, it is possible that the Pldo interaction with actin regulators might be indirect, mediated by other proteins that contain SH3 domains, like IRSp53. Interactions with SH2 and SH3 domains are important as these domains are frequently employed in proteins regulating several biological processes, including cytoskeletal rearrangements and cell migration, for example (Tatárová et al, 2012; and rev. in Kaneko et al [2008] and Mayer [2015). The physiological relevance of proteins with these domains is described in podocytes, where actin reorganization is mediated by interactions of NCK1 SH3 domain to N-WASP and NCK1 SH2 domain to phosphorylated Nephrin (Jones et al, 2006), and consequently, these interactions will regulate Arp2/3 complex activity. Of note, most of the SH2-binding motifs in Pldo are located at the N-terminal region of the protein, whereas the SH3-binding motifs are mainly found on the C-terminal portion. Such segregation could explain the different behavior observed in rescue experiments in hemocytes and hair formation with full-length Pldo and Pldo^ΔC^, suggesting differential regulation of distinct parts of the Pldo protein.

Along the above lines, the Pldo sequence reveals the presence of multiple serine/threonine phosphorylation sites, some of them clustered, and potential targets of different kinases, adding another level of context dependent regulation of Pldo. Several PCP proteins share this feature with Pldo. For example Vang, which requires phosphorylation for its polarized asymmetric localization, is phosphorylated on such serine/threonine clusters (Gao et al, 2011; Kelly et al, 2016). Similarly, Disheveled is heavily phosphorylated to channel its function to either canonical Wnt-signaling, PCP, or other functions, and it has been suggested that it contains a “barcode” of phosphorylation and that its activity is regulated in this manner (Bernatik et al, 2011; Yanfeng et al, 2011; Bernatík et al, 2014; Weber & Mlodzik, 2017; Hanáková et al, 2019). These examples suggest that PCP proteins share phosphorylation regulation, and thus, Pldo might participate in such regulatory input as a putative effector of the PCP pathway.

Phosphorylation could thus add a layer of regulation of Pldo, to both affect its subcellular function/requirement with, for example regulatory input missing in Pldo^ΔC^ and hence its lack of activity in the hair formation rescue assay (despite the fact that Pldo^ΔC^ was still very potent to induce filopodia formation in hemocytes). Similarly, phosphorylation might regulate the function of Pldo/ZSWIM8 in either actin dynamics or ubiquitin ligase activity contexts (see below).

### Pldo/ZSWIM8 does not act as part of a ubiquitination complex in actin regulation

ZSWIM8 (EBAX-1 in *C. elegans*) has been characterized as an E3 ligase and a component of a ubiquitin ligase complex (Wang et al, 2013; Shi et al, 2020), and thus, we tested whether Pldo/ZSWIM8 shares the same function in the context of actin filament dynamics. Because a *scar* knockdown reversed the *pldo/zswim8* LOF phenotype, Scar seemed to be a potential degradation target of Pldo/ZSWIM8 in the actin context. However, we did not detect any changes to Scar levels, although Scar and Pldo/ZSWIM8 are found in the same molecular complex (as seen via bidirectional IP experiments). Scar levels were also evaluated by Western blot in the presence or absence of the proteasome inhibitor MG132 and with or without coexpression of Pldo, revealing, however, no difference in any of these scenarios (whereas control proteins, like p53, showed protein-level differences). These studies demonstrate that Pldo is not regulating Scar levels. To further address this, we analyzed the effect of knockdown of components of the ubiquitin ligase complex associated with Pldo described in *C. elegans*, EloB, EloC, Cul-2, and the potential E2 ligase CG4080. None of these, assayed in LOF experiments in adult wings, showed any defects on hair formation, indicating that the ubiquitin ligase complex activity is not required in this process.

Nonetheless, although we did not detect any evidence for a ubiquitin-mediated degradation function of Pldo/ZSWIM8 in the actin cytoskeleton regulation context, we cannot rule out that Pldo/ZSWIM8 might have a function as a ubiquitin ligase in a different context. It will be interesting to determine potential regulatory conditions and mechanisms that select one function of Pldo/ZSWIM8 over the other or if there is a cell type or stage specificity to potential distinct molecular functions of this interesting protein family.

### Pldo/ZSWIM8 and a potential role in human disease

As suggested by Yamamoto, Bellen, and colleagues (Yamamoto et al, 2014), Pldo/ZSWIM8 appears to be linked to a genetic disorder in humans affecting the nervous system. This observation could be explained by our data regarding its function in the regulation of actin cytoskeletal dynamics as biogenesis of neurites requires a highly regulated actin polymerization network (Ben-Yaacov et al, 2001; Korobova & Svitkina, 2008; Matusek et al, 2008; Tahirovic et al, 2010; Yaniv et al, 2020; and rev. in Da Silva and Dotti [2002]). For example, linear actin polymerization is required for radial glial migration and in the initial stages of neurite formation and branched polymerization is essential for their maturation. As such, Pldo/ZSWIM8 levels, when altered, could easily affect this process leading to neurological defects. It is also important to consider that there are five isoforms of Pldo/ZSWIM8 in humans, making it more difficult to analyze. In addition, taken together with the data presented here, Pldo/ZSWIM8 could also have a role in cancer cells because its LOF might enhance the migratory capacity of invasive metastatic cells.

## Materials and Methods

### *Drosophila melanogaster* strains and phenotypic analysis in adult wings

*Drosophila* flies were grown on standard food at 25°C, and phenotypes were analyzed at this temperature, unless otherwise indicated.

The following fly strains were used:

Bloomington (BL): BL5137 UAS-CD8::GFP,
BL56751 UAS-Diaphanous, BL7310 UAS-Actin,
BL18553 UAS-dsRNA *pldo*,
BL52333 *pldo*^*A*^,
BL52334 *pldo*^*B*^,
BL41552 UAS-dsRNA GFP,
BL1709 FRT19A,
BL34523 UAS-dsRNA *chic*,
BL5905 *w*^*1118*^, BL24651 UAS-Dicer^2^,
BL4854 UAS-Cdc42^G12V^.

#### VDRC strains (UAS-dsRNA constructs)

21,908 *scar*, 13,759 *wasp*, 41,514 *mwh*, 102,759 *chic*, 27,623 *quail*, 101,438 *actin*, 9,026 *CG4080*, 12,953 and 101,542 *elonginB*, 15,303 and 105,740 *elonginC*, 19,297 and 105,101 *cullin2*. *UAS Scar-GFP* was a gift from S. Bogdan (Stephan et al, 2011). Overexpression of cDNA transgenes or RNAi (IR) was performed using the Gal4/UAS system (Brand & Perrimon, 1993). The Gal4 expression drivers used were as follows: *patched-Gal4*, *Hml-Gal4*, *pannier-Gal4*, and *ey-Gal4*. Where indicated, UAS-Dicer2 was included with *UAS-IR* expression to increase RNAi efficiency. All stocks employed in experiments were generated by standard crosses.

#### MARCM analysis

For pupal wing clone generation, offspring were subjected to heat shock (at 37°C) for 1 h at 80 ± 12 h after egg-laying. Pupal clones were thus marked by GFP expression. Third instar larvae possessing clones (GFP positive) were selected and determined the 0 time (white prepupae) to proceed with immunofluorescence stainings at specific times during animal development (and hair formation).

In hemocytes, the offspring were subjected to heat shock at 48, 72, and 96 h after egg laying for 1 h, then incubated at 18°C for 1 h.

For analysis of adult wing trichomes, adult wings were removed, incubated in wash buffer (PBS and 0.1% Triton X-100), and mounted on a slide in 80% glycerol in PBS.

In the trichome rescue assay, classification of the phenotypic severity of the absence of hairs in adult wings was performed by visual inspection. Criteria for classification were the extent of area without hairs. Phenotypes were classified as rescued (no area without hairs), partial rescue (small area without hairs), and no rescue (large area without hairs).

To analyze trichomes in adult nota (dorsal thorax), flies were partially dissected, incubated at 95°C in 10% KOH for 10 min to clear fat tissue, washed (PBS and 0.1% Triton X-100), and then placed in 80% glycerol in PBS. Nota were fully dissected and mounted on a slide in 80% glycerol in PBS. Adult wings and nota were imaged at RT on a Axioplan (Carl Zeiss) microscope. Images were acquired with a Zeiss AxioCam (Color type 412-312) and AxioCam software.

### Sequence analysis

Sequence analysis and predictions were made at online programs Psipred and ELM, the eukaryotic linear motif resource for functional sites in proteins.

### Immunofluorescence

For analysis in pupal wings, white pupae were collected (0 h APF) and aged at 25°C until dissection. Dissections were performed as follows: in brief, pupae were immobilized on double-sided tape, removed from the pupal case, and placed into PBS, in which pupae were partially dissected to remove fat tissue, fixed in 4% paraformaldehyde in PBS for 45 min at RT or overnight at 4°C, and washed 3× in PBS and 0.1% Triton X-100. Wing membranes were removed, and immunostaining was performed by standard techniques. In brief, tissue was incubated in wash buffer containing 10% normal goat serum overnight for primary antibody (4°C), washed 3× with PBS, and incubated with the secondary antibody for at least 2 h (25°C) and fluorescent phalloidin for staining the actin cytoskeleton. Wings were washed 3× with PBS and mounted in Mowiol. Pupal-wing images were acquired at RT using a confocal microscope, either Zeiss LSM 710 or Leica SP8. Images were processed with ImageJ (National Institute of Health).

For hemocyte immunostaining, primary culture was performed as described (Tirouvanziam et al, 2004). Briefly, third instar larvae were washed, and a small incision on the posterior side of larvae was made to obtain hemocytes, which were incubated for 1 h 15 min, fixed, and stained. Samples were maintained in VECTASHIELD until pictures were taken. Confocal images were captured using either a Zeiss LSM 710 or a Leica SP8 confocal microscopes. Images were processed using ImageJ.

### Antibodies

The following antibodies were used:

mouse anti-Fmi (1:10; Developmental Studies Hybridoma Bank),
mouse anti-Fascin (1:10; Developmental Studies Hybridoma Bank),
mouse anti-Scar (1:50; Developmental Studies Hybridoma Bank),
rabbit anti-Dia (1:1,000, donated by P Adler),
mouse anti-Chic (1:10; Developmental Studies Hybridoma Bank),
rabbit anti-PatJ (1:500), mouse anti-HA (1:50; BioLegend),
mouse anti-α-actin (1:1,000; Sigma-Aldrich).

All fluorophore-conjugated secondary antibodies were used at 1:200 and obtained from Jackson ImmunoResearch Laboratories, Inc. Rhodamine-phalloidin was from Invitrogen and used at 1:400.

### Image analysis

Individual channels from color images were converted to gray scale using ImageJ. Fluorescent intensity levels were measured on maximal projections of image stacks using ImageJ.

### DNA construct generation

Pelado ΔC was generated by PCR using the following primers: Fw: 5′-CAC CAT GGA CCG CTT CAG CTT CG-3′, Rv 1: 5′-CTC TCC TCG CGT CTT AAA GGT TCC-3′, RV 2: 5′-GAA AAT AAA CGT GGC CAG GTT AAT AGG CAA TAG-3′, RV 3: 5′-GCT GAG TGT AAC TAG GGC ATC GAA AAT AAA CGT GGC-3′. The PCR product was purified and cloned into a pENTR vector, sequenced, and using the Gateway kit, was cloned into the pUASt vector, with the coding sequence of HA tag at the 5′ of *pldo* gene. Transgenesis was performed at BestGene. The deletions Pldo^1-685^ and Pldo^1-515^ were generated by restriction enzymes KflI and BsiWI, respectively. To express Pldo in mammalian cells, the coding sequence was cloned into a VVPW/BC vector (donated by L Gusella), using XbaI and EcoRI restriction sites.

### Cell culture, transfection, and scratch wound healing assay

*Drosophila* S2R+ cells were obtained directly from the Drosophila Genomics Resource Center (DGRC), Indiana. Cells were grown in Schneider’s medium, supplemented with 10% FBS and maintained at 25°C, and transfected with Effectene reagent, following standard protocols. Cells were lysed or immunostained 48 h after transfection in lysis buffer.

Human A549 cells were obtained from the Bartel Laboratory and were grown in DMEM medium, supplemented with 10% FBS and puromycin (10 μg/μl) and maintained in 5% CO_2_ at 37°C. Monoclonal cell line was generated by seeding individual cells in a 96-well plate, monitoring cells for growth, identifying individual colonies, and expanding monoclonal lines of interest. Clonal cells knocked out for *pldo/zswim8* were identified by PCR with the primers described (Shi et al, 2020). Cells were transfected with Effectene reagent, following standard protocols.

Cell cultures were plated to a confluence of 50–70% and transfected the next day. After 8–12 h, the medium was changed for fresh medium. When confluence was reached, the scratch was performed with a yellow plastic tip. Pictures were taken at times 0, 18, 22, and 24 h after the scratch was made.

For MG132 treatment, the drug was used from a stock at 1 mg/ml, and the final concentration in each well was 10 μM. MG132 was added 36 h after transfection and maintained for 6 h. After 6-h incubation with the drug, media was changed and proceeded to protein extraction.

### Western blotting and immunoprecipitation

S2R+ cells (grown in Schneider medium using standard procedures) were transfected with HAPldo and GFP-Scar, using Effectene reagent. After 48 h, protein extraction was performed. Immunoprecipitation (IP) and co-IP experiments were performed by incubating lysates with anti-HA or anti-GFP antibody, at 4°C overnight followed by Agarose-A beads incubation, 5× washing steps, and elution in SDS sample buffer. Samples were boiled at 95°C for 10 min and proceeded to Western blotting.

### Statistical analyses

Quantifications were made in ImageJ. To determine statistical differences between data sets of continuous data, we performed nonparametric ANOVA or *t* test analyses on GraphPad. Specific analysis is indicated each time, and *P*-values lower than 0.05 were considered significant. Significance is indicated by asterisks. Sample sizes are described for each experiment in the figure legends. All experiments were performed on a minimum of three animals per condition. The statistical parameters are reported in figure legends. The error bars represent the SEM.

## Supplementary Material

Reviewer comments
