# Supplementary figures and images for: The conserved Pelado/ZSWIM8 protein regulates actin dynamics by promoting linear actin filament polymerization
Source: Life Sci Alliance. 2022 Aug 8;5(12):e202201484. doi: 10.26508/lsa.202201484 (PMC9375228; doi:10.26508/lsa.202201484)

Figure 6

Scar

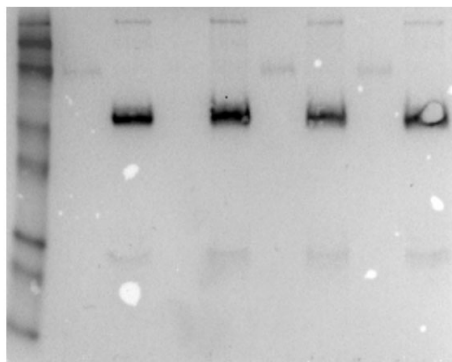

Dia

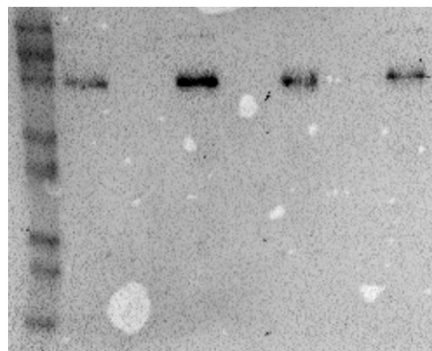

Actin

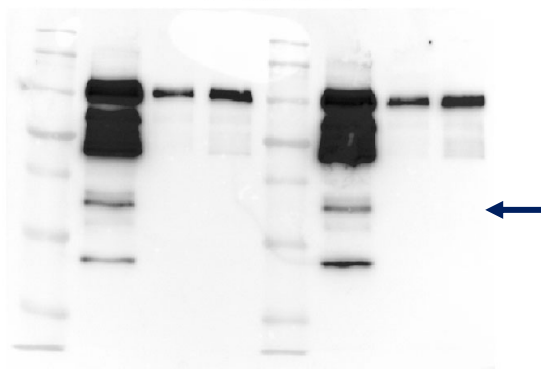

Profilin

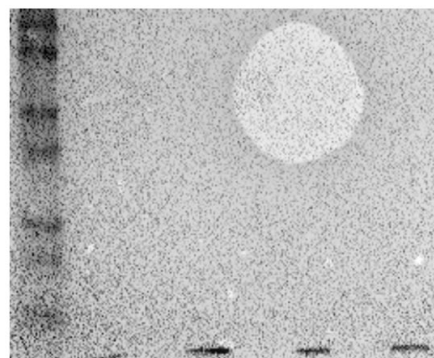

Suppl. Figure S6

Scar

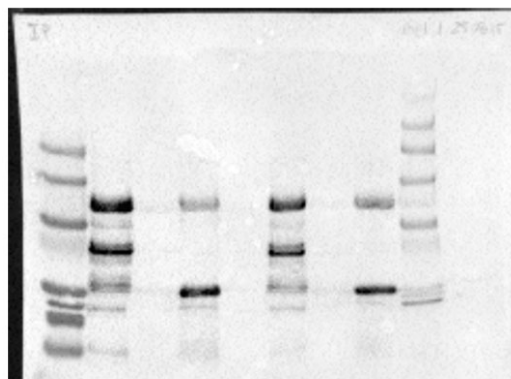

HA

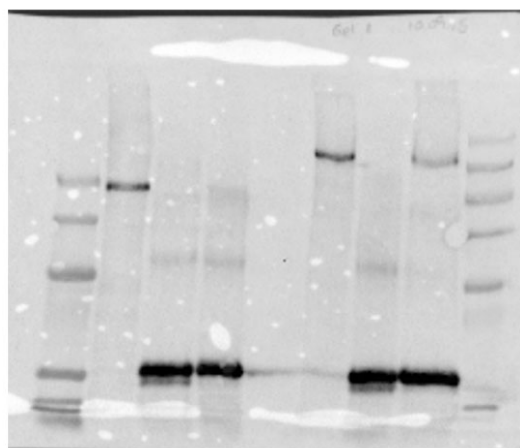

Supplement: Supplementary file 1 [file LSA-2022-01484_SdataF6_FS6.pdf]
